# Merging Real-Time NIR and Process Parameter Measurements in a Fluidized Bed Granulation Process to Predict Particle Size

**DOI:** 10.3390/pharmaceutics17060720

**Published:** 2025-05-29

**Authors:** Ozren Jovic, Marcus O’Mahony, Samuel Solomon, David Egan, Chris O’Callaghan, Caroline McCormack, Ian Jones, Patrick Cronin, Gavin M. Walker, Rabah Mouras

**Affiliations:** 1 Pharmaceutical Manufacturing Technology Centre, Bernal Institute, Department of Chemical Sciences, University of Limerick, V94 T9PX Limerick, Ireland; ozren.jovic@universityofgalway.ie (O.J.); marcus.omahony@ul.ie (M.O.); david.egan@ul.ie (D.E.); gavin.walker@ul.ie (G.M.W.); 2 Dairy Processing Technology Centre, Bernal Institute, Department of Chemical Sciences, University of Limerick, V94 T9PX Limerick, Irelandpatrick.cronin@imr.ie (P.C.); 3 InnoGlobal Technology, Ravenscourt Campus, D18 K599 Dublin, Ireland; ocallaghanc@innoglobal.com (C.O.); mccormackc@innoglobal.com (C.M.); jonesi@innoglobal.com (I.J.)

**Keywords:** fluid bed granulation, chemometrics, predictive modeling, machine learning (ML), critical quality attributes (CQAs), PLS, NIR spectroscopy, particle size, process analytical technology (PAT)

## Abstract

**Background/Objectives**: Controlling the critical quality attributes (CQAs), such as granule moisture level and particle size distribution, that impact product performance is essential for ensuring product quality in medicine manufacture. Oral solid dosage forms, such as tablets, often require appropriate powder flow for compaction and filling. Spray-dried fluidized bed granulation (FBG) is a key unit operation in the preparation of granulated powders. The determination of particle sizes in FBG using near-infrared spectroscopy (NIR) has been considered in the literature. Herein, for the first time, NIR is combined with process parameters to achieve improved prediction of the particle sizes in FBG. **Methods**: An inline model for particle size determination using both NIR and FBG process parameters was developed using the partial least square (PLS) method, or ‘merged-PLS model’. The particle size was predicted at the end point of the process, i.e., the last 10% of the particle-size data for each batch run. An additional two analyses included a merged-PLS model with 12 batches: (1) where nine batches were training and three batches were a test set; and (2) where 11 batches were training and one was a test batch. **Results**: For all considered particle size fractions, Dv10, Dv25, Dv50, Dv75, and Dv90, an improved root-mean-squared error of prediction (RMSEP) is obtained for the merged-PLS model compared to the NIR-only PLS model and compared to the process parameters alone model. Improved RMSEP is also achieved for the additional two analyses. **Conclusions**: The improved prediction performance of endpoint particle sizes by the merged-PLS model can help to enhance both the process understanding and the overall control of the FBG process.

## 1. Introduction

Fluidized bed granulation (FBG) is a well-known wet granulation (particle size enlargement) methodology in the pharmaceutical industry for preparing intermediate or active pharmaceutical products. In the FBG process, dry mixing, wetting, and drying are accomplished in a single operation unit, which simplifies the granulation process [1]. Compared to dry granulation, wet granulation offers better control of drug content uniformity, product bulk density, and compatibility [2]. Understanding the fundamental physical and chemical attributes that contribute to the granule properties and granule behavior will impact the final product. How a material granulates depends on formulation properties, equipment type, and operating conditions. Control of the fluidized bed process is essential to ensure the consistent production of granules with the desired quality characteristics (i.e., granule size, size distribution, moisture content, flowability, etc.) [1]. Batch non-conformance detection [3], accurate moisture [3,4], and granule particle size determination [5] are crucial aspects of FBG control, especially on or before the endpoint phase of the process [6]. It is therefore vital to monitor granule particle sizes to ensure the final quality of the product [5], while the quality includes uniformity in tablet weight and consistency in hardness [7].

Determination of granule particle size using near-infrared spectroscopy (NIR) is based on spectral baseline changes with differing particle sizes [7]. An early study in the nineties correlated NIR absorbances and granule particle sizes using partial least squares [8]. Later, in 2005, NIR spectroscopy was installed through a window to monitor particle size and moisture content in real time to predict the granulation endpoint [9]. NIR prediction correlated with offline image analysis (0.52 < R2 < 0.83), but the approach to be calibrated was conflated with the NIR measurement of the moisture content and could not target the particle content with high accuracy [9]. Nieuwmeyer et al. used laser diffraction as an offline method and an NIR probe for the prediction of particle sizes [10]. Particle sizes were predicted with the PLS regression method. PLS regression is a linear regression methodology that maximizes the covariance between the independent variable data matrix, X (in this case, NIR spectra (and additional process variables)) and the dependent variable vector Y (in this case, LOD or granule size) with regression coefficient vector B; Y = XB + E (where E is the residual error term). It reduces the data dimensionality from tens to hundreds of mutually correlated variables to only several mutually uncorrelated latent variables [11]. Such an approach is mostly successful for granule sizes with median diameters between 300 and 800 μm, although smaller particle sizes are predicted less accurately [10]. In 2019, Pauli et al. were successful in the real-time monitoring of particle sizes using inline and offline NIR combined with PLS while measuring the particle sizes with dynamic image analysis with a Camsizer XT [5]. They obtained the final result of median particle sizes, a root mean squared error of prediction (RMSEP) on the internal validation set of 97 μm. The authors utilized NIR wavelengths (only) as independent variables, and the result coincided with the cross-validation results (RMSECV = 96 μm). So, particle size data (Dv10, Dv50, and Dv90) with Camsizer are proven to be accurate by an offline method [5]. At the same time, Camsizer data have also been shown to be highly correlated with Eyecon (real-time particle size and shape data analyzer, InnoGlobal, Dublin, Ireland) particle size data, and it has been shown that the differences between these two inline methodologies are mostly negligible [12]. It has also been shown that with the additional feedback control across key process parameters throughout the whole process, self-guided control of an FBG process can be established, which leads to a narrower, more controlled particle size distribution at the batch endpoint [13].

Besides NIR, the granulation design of experiments was conducted by inline collected spatial filter velocimetry (SFV) data [14]. SFV measurements were sensitive to particle size change during the FBG. A span of different models was built to relate the granule tapped density and the Hausner ratio to the inline measured particle sizes [14]. A batch model was later developed to statistically control FBG based on inline collected SFV particle sizes, the product temperature, and the batch process time [15]. The developed model enabled the real-time evaluation and acceptance or rejection of test batches. Multivariate statistical control methods based on SFV measurements were also developed by Huang et al. [16] for an Aeromatic fluid bed granulator capable of manufacturing 35 kg batches. But neither of these approaches considered NIR wavelengths in their prediction models of granule sizes.

So, statistical models based on inline batch outlier detection have been built, and they comprise at least one of the fluidized bed process parameters [1,15]. In controlling the moisture level and the particle sizes, the critical process parameters, such as the spray rate, atomizing pressure, and airflow rate, are dynamically controlled to ensure target particle size data [12]. However, when predicting particle sizes, no statistical model has been built to date that considers both the NIR wavelengths and many fluidized bed process parameters. Only NIR wavelengths have been used in many studies [5,7,8,9,10], while in a separate study, only the process parameters (e.g., the flow rate and absolute humidity of inlet and outlet air) were considered [17]. For that reason, it is important to investigate the influence of fluidized bed process parameters on the prediction capability of particle sizes; if they are merged with NIR wavelengths as independent variables, the process parameters might improve the prediction model when compared to the benchmark model using NIR wavelengths (only). This analysis, by testing the merged (NIR + process parameters (i.e., NIR + pars, later in the text)) model on the prediction of granule sizes, has not been conducted in a 30-year-long publication record on the use of spectroscopy in FBG, to the best of our knowledge. If the merged (NIR + pars) model significantly outperforms the NIR model in prediction, this information might improve both our understanding of the FBG process and the control of the same process. In particular, such an analysis would (1) identify the key process parameters that enhance the prediction model and other less informative process parameters; (2) enable an approximate inline prediction of granule sizes for the data, part of the data, or part of the batch, avoiding use of an inline particle analyzer; and (3) if an inline image analyzer is used, then batches with high discrepancy between the model predicted granule sizes at the endpoint phase and the analyzer-determined granule sizes would be identified as inline determined batch outliers [18].

Also, an outlier analysis using a probability-based outlier-detection method and at least one benchmark methodology from Ref. [18] still has not been carried out on the already obtained all-batch, i.e., the offline NIR data of FBG batch systems. It is necessary to carry out such an outlier analysis, as batch failures are common and next to impossible to accurately establish using an offline methodology [15].

Regarding the literature on cross-validation errors in the prediction of Dv50 particle sizes, as differences from target values, they vary from 70.4 μm [10] to 96 μm [5], with a target value range of 48–1052 μm in Ref. [10] and 98–1017 μm in Ref. [5]. These errors are comparable to the reported laboratory reference Camsizer standard error of ±59 μm for Dv50 [5]. However, these literature RMSE values were obtained by internal validation, and the models comprise every batch (at least in part); so, they are not necessarily an estimate of a prediction error forecast of granule sizes for a new independent batch. Also, the endpoint of the granulation process is based on these RMSE values, and it does not regard the prediction of the last segment of the FBG batch experiment (e.g., the last 5% of the batch running-time data). The last drying segment most closely represents the product particle size outcome. Significantly improving the prediction accuracy of the granule particle size in this stage of the process could lead to an enhancement of fluidized bed process control. This advancement would consider inline batch granule size determination and the prediction of the whole test batch without Eyecon sizes, using previously obtained training batches with Eyecon sizes. This is the primary task of this study. The secondary task might be to identify the outlier batches and to compare them with non-outliers in the context of both the offline (i.e., already obtained all-batch data) and inline (i.e., real-time in-process) data of FBG experiments.

## 2. Materials and Methods

### 2.1. Materials

Lactose monohydrate (LM) and polyvinylpyrrolidone K90 (PVP) were obtained from Baoji Guokang Biotechnology, Qingdao, China. Microcrystalline cellulose type 101 (MCC) was bought from Pharmatrans-Sanaq^®^ AG, Allschwil-Basel, Switzerland.

### 2.2. Fluidized Bed Granulation

Fluidized bed granulation (FBG) was conducted using a Glatt Multilab unit. The setup of the unit comprises an enclosure, heater, fans, etc., used for spray granulation and drying. Control of the fluidized bed main operating ranges was implemented with and without a mechanistic model control via the process automation platform SmartX (InnoGlobal), developed for pharmaceutical fluid bed operations and process development.

This set the allowable range of process parameter set points explored during the batches to prepare the different particle sizes of granules. The total number of batches was 14. The min and max process parameters, the average process parameters, and the standard deviation and initial particle sizes are available in Table S1a–e of the Supplementary Materials. The FBG process was thus undertaken at varying process parameter ranges and values; for example, the airflow rate in all the batch experiments was within the limits of 18.7–36.0 m^3^/h (see definitions below). The formulation consisted of 66.67% LM and 33.33% MCC. A binder solution containing 5.4 wt% PVP was used as a granulating agent. Multiple granulation experiments were conducted, which used the same materials, binder solution, and loading of powders. For each experiment, a machine warming step was first undertaken, after which the powders were loaded into the product bowl of the FBG, and then warming of the powders was undertaken. Once these steps were completed, the actual granulation process was initiated by controlling the inlet airflow rate to fluidize the powders and by spraying the binder solution onto the powder bed. During the drying step, spraying of the binder solution was stopped while continuing to fluidize the granules.

**Figure 1:**
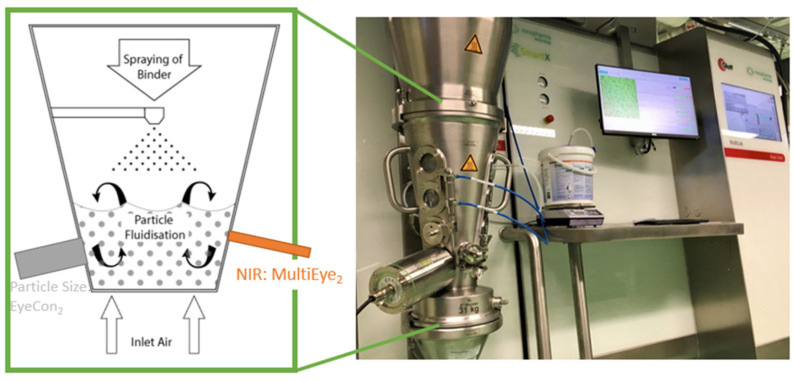
Equipment setup and a schematic of the fluidized bed spray granulation process, with an NIR and online particle size measurement system adapted from Refs. [13,19].

Figure 1 below shows the equipment setup and a schematic of the fluidized bed spray granulation, with NIR and particle size measurements in place.

The following list describes the FBG process parameters:

- Airflow rate—airflow through the entire fluidized bed, measured as the speed of air in m^3^/h.
- Atomization pressure: Compressed air pressure (in bar) at the spray nozzle to produce the droplets that enter the fluidized bed chamber.
- Exhaust air pressure—air pressure leaving the fluidized bed above the granulation area.
- Exhaust air temperature—air temperature leaving the fluidized bed chamber in °C.
- Inlet air temperature—air temperature entering the fluidized bed chamber.
- Plenum air pressure—air pressure at the lower plenum point before the fluidized bed chamber.
- Product and filter delta P mbar—the difference in pressure between the fluidized bed chamber and the filter after the chamber.
- Product temperature—temperature of the granulation product in the chamber.
- Spray-rate average—a measure of the weight of slurry that has been deposited in the fluidized bed chamber in g/min (based on the weight loss of the scales over time).
- Pressure–humidity (absolute and relative)–temperature (PHT)—sensor at the inlet (In)–outlet (Out) air point that measures absolute humidity (HuA), relative humidity (HuR), pressure (p), and temperature (T). More detailed definitions can be found below Table S1.
- Granule size (Dv90, Dv75, Dv50, Dv25, Dv10) measured by Eyecon2 given the 90th, 75th, 50th, 25th, and 10th volumetric percentile of the granule diameter. These represent dependent variables, while all prior process parameters and later NIR were independent variables in chemometric modeling.

### 2.3. Loss-on-Drying Measurements

Loss on drying (LOD) was carried out using a moisture analyzer (Sartorius MA150, Sartorius, Germany). To determine LOD, samples of ca. 1 g were used in a gentle drying mode at 110 °C. A total of 174 LOD measurements were obtained from 14 batches, and their distribution across the batches is given in Table S1f. LOD measurements are dependent variables predicted by NIR variables (see next section).

### 2.4. Process Analytical Technology (PAT)

Multieye2 is a multichannel NIR spectrophotometer designed for the rapid, real-time inline monitoring of moisture levels and granule sizes. In this study, a single NIR probe was externally mounted to a process window located within the down bed (see Figure 1 schematic). Two averaged spectra corrected against the background were recorded in the region of 1081–2122 nm every few seconds. The moisture content was obtained by building an NIR PLS model on a curated dataset (i.e., on a dataset void of two outlier batches), containing 150 data points in total, with 120 LOD data points used for building a calibration model and 30 test measurements for validating it. For granule size models, a total of 21,955 NIR spectra and granule size data points were obtained. In-depth statistical analysis is given below in Section 2.5.

Eyecon2, a non-product contact direct-imaging particle size analyzer, was utilized for inline particle size measurements. It was positioned on the outside of a process window, located within the down bed to capture representative data (see Figure 1). To mitigate window fouling, a mechanical wiper/scraper prototype was installed and configured to periodically clear the inside of the window.

### 2.5. Chemometrics

#### 2.5.1. Data Preprocessing

The scaling of all variables was conducted except for the target variables (i.e., LOD and granule sizes) in supervised partial least squares (PLS) modeling to predict the actual values of the target variables. In the application of unsupervised principal component analysis (PCA) to the datasets, all the variables, including the LOD and granule sizes, were scaled to be able to compare variances directly between NIR, the process parameters, and the target variables.

#### 2.5.2. PCA Procedure for Outlier Detection and Process Parameter Covariance

PCA was carried out on all 174 NIR spectra only (NIR-PCA), with each spectrum containing its corresponding LOD value. PCA was also carried out on the scaled process parameters (SPP-PCA). The task of both NIR-PCA and SPP-PCA was to investigate the batches whose NIR spectra appeared as outliers with the potential for covariance in specific process parameters in those batches. In addition, a combination of NIR-PCA and SPP-PCA was conducted, utilizing the scores of the first four NIR-PCs and the SPPs as input for the new PCA. We also calculated the PCA-Mahalanobis distance metric and probabilistic-based metric for outlier detection [18]. To further determine outliers, soft independent modeling of class analogy (SIMCA) [20] was performed on the squared Mahalanobis distances to obtain the t statistic for the non-outlier class.

#### 2.5.3. PLS Procedure for Outlier Batch Detection

PLS modeling was performed using the NIR spectra as input and LOD as the target response data to determine outlier batches using PLS cross-validated with backward batch elimination (BBE-PLS). In this procedure, all (174) samples were cross-validated, and the root mean square error of cross-validation, or RMSECV, was obtained for all the batches. Then, in the first cycle, only one batch was removed, and leave-one-out cross-validation (RMSECV) was calculated for the rest. This was repeated so that the elimination of each batch generated an updated RMSECV for that model set in the absence of the eliminated batch. The outlier batch identified then was that for which eliminating that batch led to the lowest RMSECV among all the other single batch eliminations, and that such a change in RMSECV due to the elimination of that batch was statistically significant when compared to RMSECV containing all batches. A significance test was performed using a one-tailed t-test of equal variance on squared cross-validated residuals, with a *p* < 0.1 significance level. If the test was significant, the second batch elimination cycle was carried out by eliminating the next batch, with this removal producing the lowest RMSECV among all the remaining batches. This procedure was repeated until the last cycle, in which the lowest RMSECV was insignificant when compared to the prior elimination cycle. All batches that remained in the last cycle were non-outlier batches, while the batches eliminated in the prior cycles were outlier batches according to the BBE-PLS on the scaled NIR data. This BBE-PLS procedure can be automated for any new batch for outlier detection purposes. The task of both the PCA and BBE-PLS was objective outlier batch identification.

For the final batch prediction dataset with outlier batches removed, 120 (out of 150) non-outlier samples were training samples, while the rest were test samples. This train–test split was a sequential split, with one-fifth being the test samples and all the other samples being training samples.

### 2.6. Granule Size Prediction Procedure Using PLS

**Figure 2:**
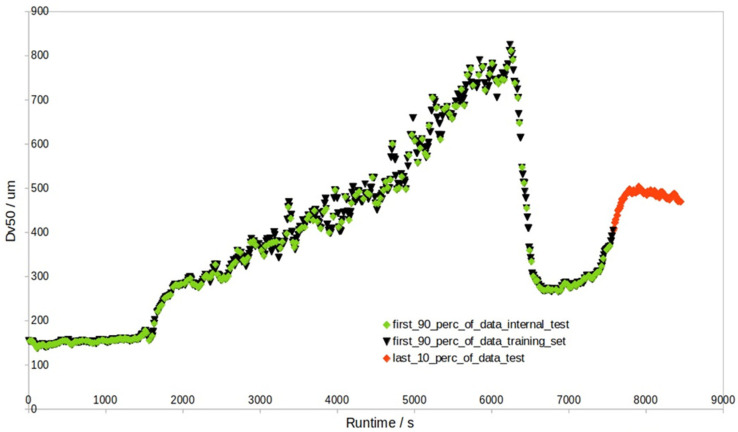
Example batch ‘1005’ contains 2644 data points. The last 10% (i.e., the last 264 red points) represent the external test set, while the first 90% are divided into a training set (sequential black 9/10 split) and an internal test set (green 1/10).

In the granule size data batches, different modeling approaches (MA) were considered and implemented. The first MA (MA 1) was inline granule size prediction, where the first 90% of the individual batch data were used for the prediction of the last 10% of the individual batch data (i.e., external test set), and in the first 90% of the data, 1/10 were also test samples (but internal test samples) in sequential order (see Figure 2 for details).

Only one batch was used for the prediction of itself, and this was the case for all batches in this MA. The second MA (MA 2) considered the use of nine batches to predict the granule sizes of the rest three non-outlier test batches and then repeated that so that all the non-outlier batches were predicted at least once as test batches. This MA was repeated, but instead of nine batches predicting three (MA 2a), eleven batches predicted one, and then repeated that cycle in a cross-validation manner so that all the batches were predicted once (MA 2b). An additional MA was performed; it was almost the same as MA 1 but regarded the test prediction of granule sizes for the last 50% of the last zero spray-rate cycle (which is when the spray rate drops to zero). Only the last part of the data formed the external test set; the rest were a training set (90%) and a sequential internal test set (10%), as in Figure 2. It also used one batch for the prediction of itself, and this was the case for all batches in this MA (MA 1-add). We also considered an additional MA 2c (eight batches predicting four non-outlier batches), and the results are reported in the Supplementary Materials. Lastly, we built the MA 1 and MA 2 prediction models using 17 process parameters only (Pars-only model) to compare the performance of these models with the NIR + pars model.

Besides ordinary PLS, uninformative-variable elimination PLS (UVE-PLS) [21] was considered and carried out to determine the significant variables. The stability threshold was determined using a minimum RMSECV [22] but with consideration of 20 different linearly incremented cutoffs, with the maximum being (nLVs + 2), the top stability value (see the entire UVE-PLS code at the end of the Supplementary Materials).

## 3. Results and Discussion

### 3.1. Outlier Batch Identification

**Figure 3:**
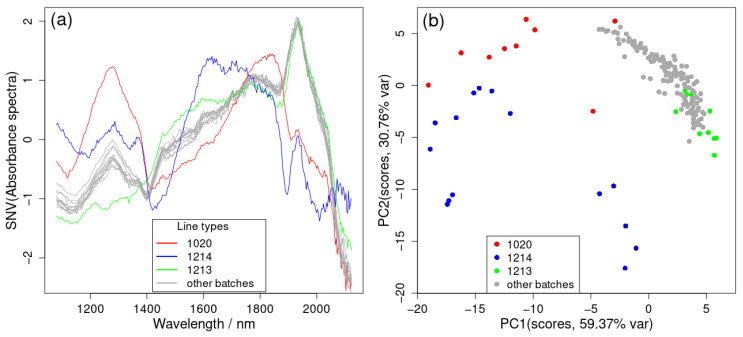
(**a**) Average SNV of the NIR spectra for each of the 14 batches, and (**b**) first two principal components (accounting for 90% of the total variance) of the SNV of the NIR spectra for all LOD-NIR data batches. The 3D plot can be found in Supplementary Figure S1a in the Supplementary Materials.

Figure 3a displays the average NIR spectra for all 14 batches of the samples that contain LOD measurements (in further text, LOD-batches). It can be observed that some batches ‘1020’, ‘1214’, and ‘1213’, severely deviate from others. Figure 3b presents a plot of the first two principal components (PCs), which account for 90% of the data variance. From here, it can be seen that all the ‘1214’ batch spectra are separated from other batch spectra, and almost all from the ‘1020’ batch. On the other hand, most of the ‘1213’ batches fall within the range of other batch spectra. According to Supplementary Figure S1a, all the samples from the ‘1020’ and ‘1214’ batches are classified into two separate clusters from the rest of the NIR SNV spectra using the first three PCs. By using a combination of the plot of the explained variance number for each PC and also referring in part to the Kaiser rule (selection of the number of components with eigenvalues > 1) [23], the optimum number of PCs was determined to be five for the NIR spectra. The strict Kaiser rule would give seven, but Kaiser scores (obtained as wavelength variables (256) times the fraction of explained variance) for the sixth and seventh equal only 1.1 and 1.03 and are smaller when compared to the fourth (4.77) and fifth (3.44). As the Kaiser rule in the literature mostly overestimates the number of PCs [24], five components were selected to be significant. This is well illustrated in Figure S1b, displaying a ln(Eigenvalue) vs. the number of components scree plot, from which it is obvious why five components is the optimal choice.

**Figure 4:**
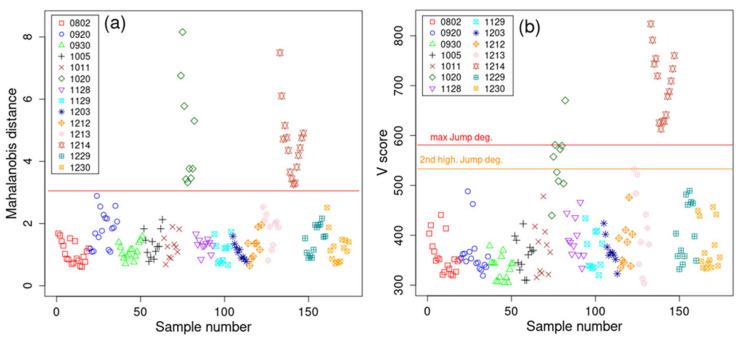
(**a**) Mahalanobis distance using the first five PCs of the SNVs of the NIR spectra for each of the 14 batches, and (**b**) V score based on Ref. [18] calculated using the SNVs of the NIR spectra. Here, the maximum jump threshold and the second highest threshold (close to the maximum threshold) were considered.

From Figure 4a, it can be observed that all the NIR spectra from both the ‘1020’ and ‘1214’ batches are obvious outliers. To be more objective, from the squared Mahalanobis distances (using a *t*-test), soft independent modeling of class analogy (SIMCA) [20] revealed the *p*-values for all the non-outlier batches (i.e., all the samples below the red line in Figure 4a). The closest sample below the red line (for batch ‘0920’) had *p* < 0.001, and the next four closest samples had *p* < 0.01. This means that all the samples above the red line (with Mahalanobis threshold = 3) have a *p*-value far below 0.001 and should be classified as outliers. Also, if less than five PCs were considered, e.g., according to the scree plot, three PCs, then Figure S2 represents the Mahalanobis distance metric of the first three PCs applied to all the NIR spectra, making the outlier batches even more separated from the rest (see Figure S2). Figure 4b presents the results of the probability-based outlier-detection method [18], and the two highest jump degrees were considered, as they differ negligibly in size. From the figure, it can be seen that all the ‘1214’ spectra are clear outliers, while depending on the jump degree selected, either one or five outliers are present in batch ‘1020’. No samples from other batches are determined to significantly deviate from the rest.

**Figure 5:**
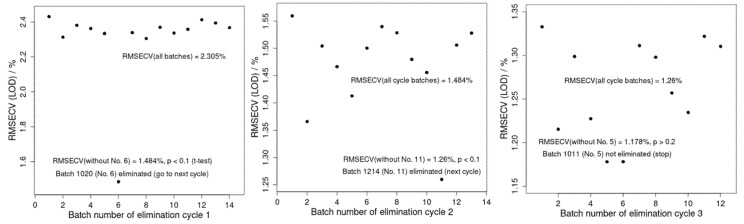
BBE-PLS procedure. The result is that batches ‘1020’ and ‘1214’ are eliminated (based on a one-tailed *t*-test, *p* < 0.1 significance level), while the other 12 are retained (*p* > 0.2).

To further prove the outlier batches, the BBE-PLS procedure for predicting LOD values was carried out as described in Section 2. The results of the BBE-PLS are presented in Figure 5. It reveals that, based on lower and significant *p*-values, both ‘1020’ (*p* < 0.1) and ‘1214’ (*p* < 0.1) are outlier batches, while other batches are not (*p* > 0.2).

**Figure 6:**
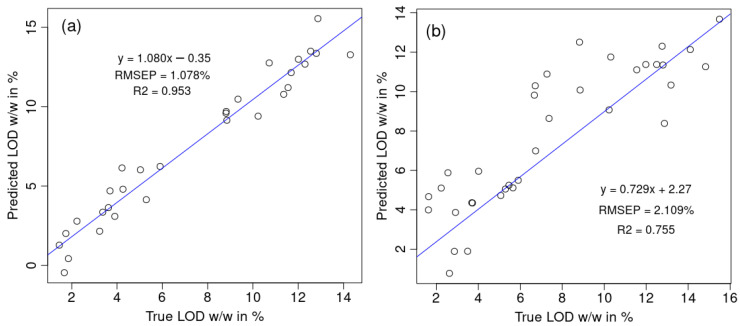
Comparison between the model’s accuracy on the test set (30 NIR spectra containing LOD values) between (**a**) the dataset without outliers and (**b**) the dataset containing outlier batches.

As LOD prediction is very important for the FBG process and already well elaborated in many published articles [6,7,25], this confirms the prior SIMCA-based analyses on outlier/non-outlier batches. Since the moisture level is calculated from the NIR spectra, outlier batches have lower capability for estimating LOD. When the outlier batches are removed, the obtained model performance of the 30 test spectra, RMSEP (LOD) = 1.078% (Figure 6a), is comparable with the one already obtained in the FBG literature on moisture quantification, RMSEP = 0.877%, of the 13 displayed data points in Figure 5b in Ref. [3]. Our obtained calibration results for 120 spectra are R2(cal) = 0.936, RMSEC = 1.064, and RMSECV = 1.347, which are again comparable with the calibration results of Ref. [3]: R2 = 0.943, SEC = 0.999, and SECV = 1.090 for only 28 displayed points in Figure 5a in Ref. [3]. Our model without outlier batches (Figure 6a) significantly outperforms the model containing outliers (Figure 6b).

**Figure 7:**
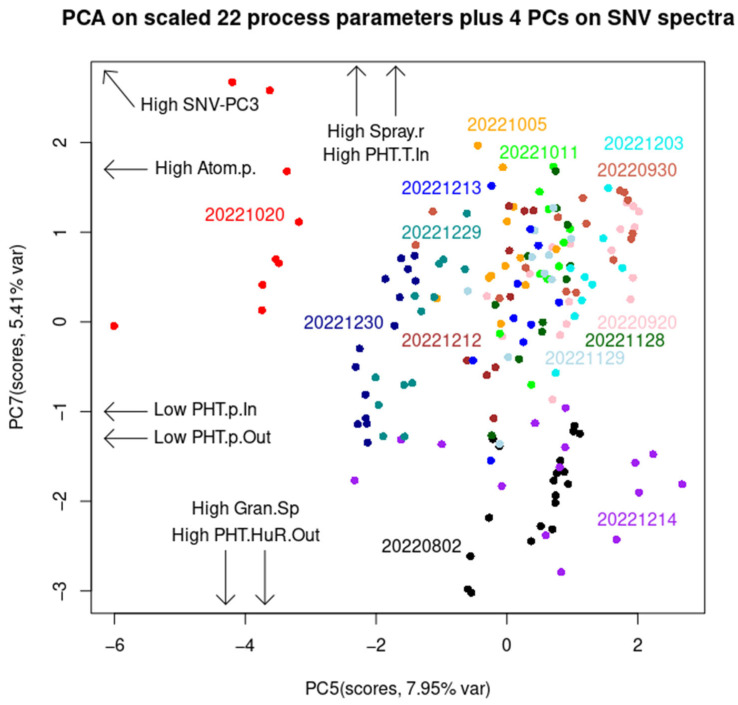
Combination PCA (of NIR (1–4 PCs) and SPP PCA). As can be seen, batches ‘1020’ and ‘1214’ have mutually opposite extremes regarding spray rate, atomization pressure, and other depicted process parameters. Between them are other non-outlier batches. Other PC scores and loading vectors can be found in Supplementary Figures S3–S7.

Figure 7 displays a score plot with arrows denoting the direction of certain process parameters. Here, PC5 and PC7, although with only a 13.1% total variance, contribute to discriminating between the batches ‘1020’ and ‘1214’. This is not too unusual, as these two batches contain 24 out of the total 174 LOD measurements, and 24 divided by 174 equals 13.7%. These two batches differ from other batches in many different process parameters and in the opposite way. Concretely (Figures S3–S7, Tables S1–S4), batch ‘1020’ has the highest average values for the following process parameters among other batches: LOD, airflow rate, atomization pressure, exhaust air temperature, inlet air temperature, plenum air temperature, product temperature, spray rate, and PHT pressure inlet. All ‘1020’ batch samples have a higher atomization pressure than the rest of the samples in all the other batches. The ‘1020’ batch samples also have the lowest PHT pressure inlet and outlet and the lowest endpoint Dv50 granule size of only 75 μm. On the other hand, batch ‘1214’ has the highest endpoint Dv50 granule size of 511.7 μm, in conjunction with other process parameters of opposite extremes to those in batch ‘1020’ (Figure 7). For batch ‘1020’, there is no significant correlation between the SNV-PC1, SNV-PC2, and SNV-PC3 scores and any process parameter. In contrast, batch ‘1214’ has a very high positive correlation between SNV-PC1 and the PHT humidity inlet (r = +0.93) and outlet (r = +0.96). SNV-PC1 represents water content, while high SNV-PC3 is followed by a high atomization pressure and spray rate, and a low PHT pressure inlet and outlet. Nevertheless, it is interesting to note that if SPP-PCA is carried out alone (i.e., without the NIR spectra), this analysis would barely classify batch ‘1020’ (Figure S6) and would not discriminate ‘1214’ from other batches (Figure S8). However, the PCA on 12 non-outlier batches was able to classify them into four groups: I. batch 0802, II. batch 0930, III. batches 1212, 1213, 1229, and 1230, and IV. the other six batches (Figures S9 and S10). This is because batch 0802 has the highest average PHT absolute humidity inlet and outlet, and PHT relative humidity inlet, while batch 0930 has the lowest average product temperature and spray rate and the highest average PHT relative humidity outlet. The simple analysis based on airflow rate, inlet air temperature, atomization pressure, and pump speed could not discriminate outliers from non-outlier batches (see Figure S11). More batch classification information is in the Supplementary Materials.

### 3.2. Prediction of Granule Sizes, NIR-PLS vs. NIR + par-PLS Comparison

Table 1 displays the Dv50 parameters for two outlier batches and the average of all non-outlier batches. From the table, it can be seen that the laboratory standard deviation equals 138.6 μm. From that, the residual predictive deviation as the ratio of that deviation to the RMSEP can be easily calculated; this is referred to as RPD (st. dev/RMSEP) in the literature and is expected to be at least 1.75, although excellent models achieve RPD > 3 [5,26]. The next observation is that the range between the max and min of Dv50 equals 824.7 − 52.4 = 772.3 μm. This, when divided by the RMSEP, is called the range ratio error (RER, RER = range/RMSEP), which is expected to be >10 [5]. The laboratory error for the 12 non-outlier batches was calculated as a standard deviation of 12 endpoint Dv50 granule sizes. This was determined to be 70.3 μm. The ratio of RMSEP to that laboratory error (i.e., RMSEP/Lab. error) is called the ratio of the prediction error to the laboratory error (PRL) and is expected to be ≤2 for good models [5]. Finally, the interquartile distance (difference between the third and the first quartile) was also calculated, and the ratio of that to the RMSEP (i.e., (Q3-Q1)/RMSEP) is called the ratio of performance to the interquartile distance (RPIQ), and very good models have RPIQ > 3 [26]. In contrast to the Residual Prediction Deviation (RPD), the RPIQ makes no assumptions about the observed value distribution [27]. RPD, RER, PRL, and RPIQ are important chemometric attributes that standardize the estimate of the model’s prediction performance, contrary to the simple RMSEP value [27,28].

**Figure 8:**
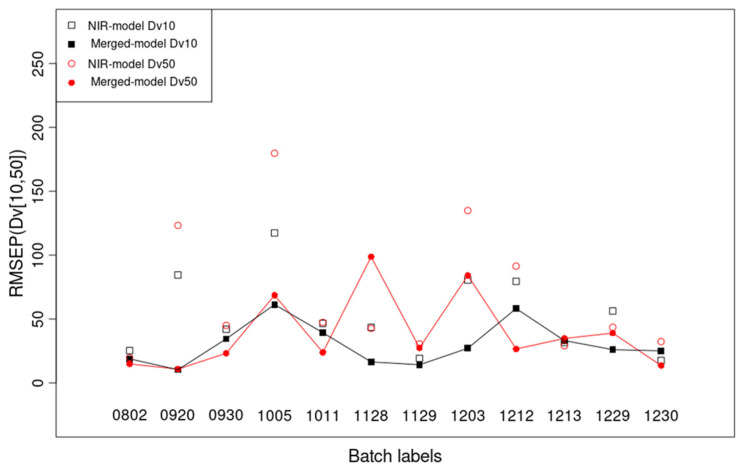
Significant difference in granule sizes (Dv10 and Dv50) between the NIR model (unfilled squares and circles) and the merged model (NIR + pars) (filled ones connected with lines) from the external test RMSEP for MA 1 (see Table S6 for full information).

Table 2 and Table S6 present the results for MA 1, described in Section 2, regarding the external test set RMSEP. From Table 2, it can be seen that all three granule-size NIR + pars models outperform the NIR (only) models. The difference between these two models is significant for granule sizes Dv10, Dv25, and Dv50 (*p* < 0.05, 1-tailed paired *t*-test) (Table S6). Moreover, if outlier batches ‘1020’ and ‘1214’ are neglected, for all the 12 non-outlier batches, the non-equality RMSEP (Dv50) < 100 μm rules for the NIR + pars model (Figure 8, Table S6). For the outlier batches, in almost all cases (except for D10 and Dv25 of the NIR + pars model), RMSEP > 100 μm, with RMSEP > 200 μm for Dv75 and Dv90. This means that using Dv50, Dv75, and Dv90 prediction statistics, outlier batches can be recognized during the inline recording of the last 10% of data, using the first 90% of data in MA 1, without the necessity of using any other batches for prediction. Worse prediction statistics for outlier batches coincide with the batch outlier detection detailed in the prior subsection. This novel information is important for future inline batch outlier detection in the FBG.

**Figure 9:**
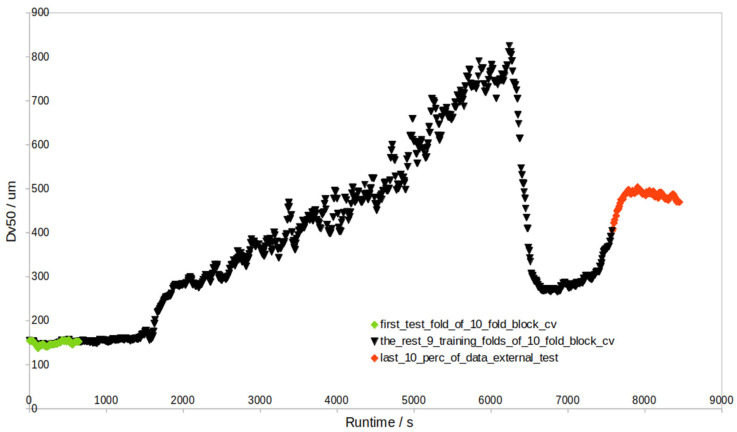
Block-CV approach (results in Table S10) on the example batch ‘1005’. The last 10% (i.e., 264 red points) represent the external test set (i.e., they are not included in CV), while the first 90% (black and green) are the training set, but CV is performed block-wise (green samples) in contrast to sequential-wise CV (Figure 2).

When the external test results in Table 2 and Table S6 are compared with those of the internal test (Table S7, Figure 2), the difference is prominent. The validation of 1/10 of the internal test data revealed much lower RMSEP values (Table S7), and the internal RMSEP highly agrees with the RMSECV of the training data (e.g., for NIR + pars-PLS with outliers, RMSECV (Dv50) = 19.7 μm, RMSEP (Dv50, internal test) = 19.3 μm). At the same time, there are no signs of overfitting, as the RMSECV/RMSEC ratio is 1.0 (Tables S8 and S9). This means that RMSEP for the internal test set cannot be simply used as an estimate of error for the endpoint granule size prediction (i.e., the last 10% data), as obviously, such an internal test set is too different from an external test set. This must be emphasized, as the internal and external test sets defined in Figure 2 have different levels of prediction difficulty. The literature on granule size prediction has not yet made any such clear distinction. This issue can be resolved by replacing the sequential-CV approach (Figure 2) with a block-CV approach (Figure 9, Table S10), as the obtained RMSECV (average Dv50 = 34.7 μm) with block-CV can roughly estimate the average RMSEP of the external test set (average Dv50 = 38.8 μm). However, the block-CV approach yields RMSECV vs. the number of component plots where either too few or too many PLS components are selected, which leads to PLS models of even worse external RMSEP (average Dv50 = 44.1 μm). This is why sequential-CV, yielding RMSECV plots with a more accurate selection of the number of components, was used most of the time in this study.

With respect to Table 2, additional prediction models were built that included the spray rate as the 18th independent process parameter variable (see NIR + pars columns in Table S11). But on average, inclusion of the spray rate slightly worsened the prediction performance for MA 1 and did not have a significant effect for MA 2, which is surprising, as one might expect the opposite, at least in relation to already published articles [12,13].

Regarding MA 1-add, which predicted the last 50% of the spray-rate data that equals zero instead of predicting the last 10% of the overall data (see columns labeled ‘50%-l.s.’ in Table S11), the results show that such an approach might only be recommended if the 50% of the zero spray-rate data comprises less than 10% of the overall data; otherwise, it is better to build prediction models with 90% of the overall data. In short, spray-rate data do not help to improve granule size model prediction performance. The same conclusion is obtained with MA 2, where the results are almost entirely the same as those without the spray-rate variable. The spray rate, in the form of the seven-point moving average, obviously does not contain beneficial information in relation to other process parameters.

Table 3 displays the results of the prediction accuracy for MA 2a, in which three batches are predicted from the nine batches. The presented results once again strongly favor the NIR + pars-PLS model rather than the NIR-PLS model. Here, it can be added that the average Dv50 RMSEP values in Table 2 and Table 3 (90.6 μm and 94.8 μm) for the NIR-PLS model seem to roughly coincide with those already published in the literature (Dv50 RMSEP = 97 μm [5] with a slightly wider Dv50 range, 98–1017 μm). Also, the average CV error obtained here, CV = 71.95 μm, appears to coincide with Ref. [10] (Dv50 RMSECV = 70.4 μm, with a slightly wider Dv50 range, 48–1052 μm). This means that our benchmark NIR (only) model realistically follows the already published literature on NIR (only) models. The NIR + pars-PLS model, being significantly more accurate, contributes to granule size prediction. Table 4 presents the final results for MA 2b, which again significantly favor the NIR + pars-PLS model. For the NIR + pars-PLS model, RMSECV = 40.92 μm was obtained, which is also a significant improvement compared to both our results and the literature results. The obtained RMSEP values outperform those in the literature [5]. The obtained average RMSEP (80.1 μm) for the NIR spectra is also not far from the literature (96 μm) value. However, the literature RMSEP of 96 μm was based on internal batch analysis, where all batches were part of both training and test sets. Although Ref. [5] considered external inline validation, it did not present the corresponding RMSEP for such predictions, only a fragmentary visual relationship between NIR-predicted and some reference PSD values. Ref. [10] also did not state that the whole independent test batch was NIR-predicted from different training batches.

In that context, Table 5 presents the final statistics in terms of important standardized attributes (RPD, RER, PRL, and RPIQ—already presented and defined in the first paragraph of Section 2.6) of our NIR-PLS and NIR + pars-PLS models. These statistical parameters confirm the efficiency of the obtained models in granule size predictions (as is already explained in the first paragraph of this section). For NIR-PLS RPD > 1.75, our study only confirms the results of Ref. [5], which also obtained RPD > 1.75. However, the NIR-PLS model performed poorly in MA 2 (RPD < 1.75). A similarly weak result for NIR was obtained regarding the RER statistic (<10) for MA 2, while again, our study reconfirms successful inline granule size predictions (RER > 10) for MA 1 [5]. RPIQ was obtained for NIR-PLS < 3, but for MA 1, there have already been reported values close to 3 for acceptable prediction models [26]. The NIR + pars-PLS model is a novel methodology, and it obtained very accurate models for MA 1 (RPD > 3, RER > 10, PRL < 2, RPIQ > 3), contrary to NIR-PLS. For MA 2a and 2b, contrary to NIR-PLS, NIR + pars-PLS obtained satisfactory prediction models (RPD > 1.75, RER > 10, and RPIQ > 3 for MA 2a and RPIQ close to 3 for MA 2b). This means that, for the first time, NIR + pars-PLS is capable of predicting whole-batch granule sizes in the potential absence of Eyecon, should all process parameters be provided, and if the batch is determined to be a non-outlier batch.

That the addition of 17 process parameters improves granule size prediction performance was also confirmed by MA 2c (Table S12). However, the importance of these 17 process parameters is not the same when improving the prediction model. The higher the absolute value of the regression coefficient is, the higher the variable importance. The overall mean for the SNV NIR wavelengths equals 1.86, and the standard deviation (STDEV) is 1.51; so, mean + 2 STDEV = 4.88 margin. Only six wavelengths have an average absolute regression coefficient above that (4.88) margin for MA 2a. These are NIR absorbances at 1934 nm, 2066 nm, 2109 nm, 1896 nm, 1740 nm, and 2069 nm. On the other hand, Table 6 displays average absolute regression coefficients for 17 process parameters sorted in decreasing order. These process parameter coefficients are much larger than the average wavelength coefficients. The most dominant is the airflow rate. However, one should recall that many NIR wavelengths are mutually highly correlated, so these higher absolute values for the 17 process parameter regression coefficients compared with the average of 256 NIR wavelengths are not so unexpected. But the highest weighting for the airflow rate in the PSD of Dv50 prediction has not yet been reported in the records for FBG processing.

Except for the airflow rate, other important process parameters given in Table 6 in decreasing order are from runtime to PHT outlet pressure. A significant contribution to the prediction performance from the runtime variable was not an expected result [15]. The effect of runtime, by repeating MA 1 and MA 2 without the runtime variable (i.e., now with 16 process variables), was further investigated, and the results can be found in Tables S13–S15. From these supplementary tables, a short conclusion can be made that it is better to omit runtime in MA1 and include it in MA2. This means that independent batches are better predicted if runtime is incorporated in the prediction model, but for the prediction of the end of the runtime of the granulation process, it is completely redundant within the one-batch MA. The last four process parameters have absolute regression coefficients below the set margin and are less important, but there are only four of them. These are product temperature, PHT inlet pressure, product and filter difference in pressure, and PHT inlet pressure. This is also a surprising result, as product temperature has already been successfully regressed vs. granule size, but in conjunction with process time and without the NIR variables for batch control in the SFV experiments [15]. The other 13 process parameters are determined to be important for the prediction of granule sizes, with airflow rate, atomizing pressure, and running time being the most important. This information has been previously unknown in literature to the best of our knowledge.

Granule size prediction models built with process parameters only (Pars-only) are already known [17], so we predicted granule sizes (Dv10, Dv25, Dv50, Dv75, and Dv90) with Pars-only-PLS (17 process parameters) using MA 1 (Tables S16–S19) and compared the prediction performances with those of the NIR + pars-PLS model (Tables S6–S9). Except for Dv10, where the difference was insignificant, for all other granule sizes (Dv25-Dv90), NIR + pars significantly outperformed the Pars-only model (*p* < 0.05). Regarding MA 2, the comparison in Tables S20–S22 reveals that for MA 2b, the difference in model performance is, on average, negligible (3.7% relative difference in RMSEP in favor of Pars-only). However, in the case of MA 2a and MA 2c, the average difference is significantly in favor of the NIR + Pars model (in both cases, 12.7% rel. diff. (RMSEP) in favor of NIR + pars). In summary, NIR + pars improves granule size prediction when compared to the Pars-only model.

Apart from (ordinary) PLS, uninformative-variable elimination PLS (UVE-PLS) [29,30] was considered, and the selection of the most important and least important variables with UVE-PLS coincided with those of PLS. Regarding accuracy, for UVE-PLS, the obtained RMSEP values in MA 2a are the following for the experiments: RMSEP = 64.03 μm (Exp 1), 81.05 μm (Exp 2), 53.95 (Exp 3), 64.64 μm (Exp 4). The average RMSEP for UVE-PLS = 65.92 μm, which is negligibly better than that of the PLS results (67.21 μm, see Table 3). UVE-PLS selected fewer variables, instead of 273 (of which 17 were process parameters), for each experiment: 68 variables for Exp. 1 (14 proc. param.), 123 for Exp. 2 (12 proc. param.), 102 for Exp. 3 (13 proc. param.), and 159 for Exp. 4 (15 proc. param.). On average, among the 273 variables, 113 (i.e., (68 + 123 + 102 + 159)/4 = 113) were determined to be important, which is 41%, but among which, on average, 13.5 ((14 + 12 + 13 + 15)/4 = 13.5) process parameters were found to be important, which is 79%. This UVE-PLS procedure on MA 2a therefore confirms the importance of including 17 process parameters when predicting granule sizes, as a larger fraction of spectral (NIR) variables are determined to be unimportant compared with a fraction of the included process parameters in the merged-PLS model. Finally, and on the other hand, relying only on process parameters (by excluding NIR variables) would not build optimal granule size prediction models, as our results also suggest.

## 4. Conclusions

In this work, for the first time, a merged-PLS model composed of 256 NIR spectral variables and 17 fluidized bed process parameters was used to predict online measured granule sizes. The target of prediction was the endpoint phase of each batch process and offline whole validation batches (1 or 3) from other training batches (11 or 9). The final results show that merged-PLS not only outperformed the NIR-PLS model but also proved the capability of the merged-PLS model for the whole of the Dv50 batch granule size prediction (based on RPD, RER, PRL, and RPIQ). The improvement in the model is due to the positive effect of at least 13 out of 17 process parameters (only 4 of them are on average less informative). Apart from Dv50, significant improvement in the model is also seen in the predicted performance of smaller granule sizes, i.e., Dv10 and Dv25. For Dv75 and Dv90, the improvement was still present for the merged-PLS model, but it was not as good as that for smaller granule sizes. Also, the merged-PLS model was superior on average to the parameter-only model. Future perspectives should consider using the merged-PLS for granule size prediction for (1) inline recognition of outlier or non-conforming batches that include active pharmaceutical ingredients (APIs) and (2) the prediction of granule sizes.

## Figures and Tables

**Table 1:** Statistics related to the experimental Eyecon Dv50 granule sizes (μm). For Dv10, Dv25, Dv75, and Dv90, see Table S5.

Batch	Start Point	Endpoint	Mean	St. Dev.	Min	Max	Range	Q3-Q1	Lab. Error
1020	202.1	75.0	379.6	180.0	64.3	661.0	596.7	309.4	-
1214	149.7	511.7	416.0	192.7	99.4	765.6	666.2	315.1	-
Non-outliers	144.5 *	300.9 *	314.0	138.6	52.4	824.7	772.3	192.2	70.3

* The average for 12 non-outlier batches.

**Table 2:** Comparison of the granule size external test RMSEP for MA 1 between the NIR-PLS model and NIR + pars-PLS (NIR+ 17-PP) (see Table S5 for full information). Experimental values are in Table 1 and Table S5.

	Dv10		Dv50		Dv90	
	NIR	NIR + par	NIR	NIR + par	NIR	NIR + par
Average *	60.4	39.4	90.6	57.9	151.6	136.7
aver. -outliers **	53.7	30.4	68.3	38.8	111.1	91.1
*t*-test 1-t, p.	0.0118		0.0171		0.326	

* Average RMSEP across all batches. ** Average RMSEP across all batches without outliers.

**Table 3:** Comparison of granule size prediction accuracy for MA 2a, i.e., for three-batch prediction data between the NIR-PLS model and NIR + pars-PLS (NIR+ 17-PP) (using nine other batches).

Test Batches	NIR Spectra Only Model				NIR Spectra + 17 Process par. Model			
	RMSECV	R^2^CV	RMSEP	R^2^te	RMSECV	R^2^CV	RMSEP	R^2^te
0930, 1129, 1229	69.90	0.732	91.45	0.649	34.87	0.933	62.63	0.858
0920, 1011, 1213	72.17	0.759	96.35	0.618	44.20	0.910	77.00	0.621
0802, 1128, 1212	68.06	0.779	77.87	0.581	40.99	0.920	56.83	0.827
1005, 1203, 1230	60.67	0.762	113.54	0.576	39.59	0.899	71.39	0.823
Average	67.7	0.758	94.80	0.606	39.91	0.916	66.96	0.782

**Table 4:** Comparison of granule size prediction accuracy for MA 2b, i.e., for one-batch prediction data, between the NIR-PLS model and NIR + pars-PLS (NIR+ 17-PP) (using eleven other batches).

Test Batches	NIR		NIR + pars	
	RMSEP	R^2^te	RMSEP	R^2^te
0802	57.53	0.811	64.19	0.861
0920	101.0	0.281	86.99	0.446
0930	117.2	0.722	76.50	0.876
1005	135.5	0.471	67.64	0.934
1011	70.81	0.840	33.88	0.947
1128	98.48	0.578	41.31	0.884
1129	49.97	0.824	40.82	0.906
1203	102.3	0.761	49.97	0.961
1212	59.42	0.797	54.10	0.847
1213	81.70	0.680	87.88	0.457
1229	40.45	0.897	62.94	0.921
1230	46.78	0.865	43.95	0.947
Average	80.10	0.711	59.18	0.832

**Table 5:** Final non-outlier result statistics for Dv50 in the external test sets.

	NIR-PLS				NIR + pars-PLS			
	RPD	RER	PRL	RPIQ	RPD	RER	PRL	RPIQ
MA 1	2.03	11.3	0.97	2.81	3.57	19.9	0.54	5.02
MA 2a	1.46	8.15	1.35	2.03	2.06	11.5	0.96	2.86
MA 2b	1.73	9.64	1.14	2.40	2.34	13.1	0.84	3.25

**Table 6:** Importance of each of the 17 process parameters in terms of absolute regression coefficients for MA 2a (which are average absolute coefficients of the four models in Table 3).

Process Parameter	Abs. Reg. Coef.
Airflow rate	68.63
Runtime	20.19
Atomizing pressure	19.77
PHT outlet temp.	13.83
Inlet air temp.	12.66
Lower plenum pres.	12.49
Exhaust. pressure	11.70
PHT In. Abs. Hum.	8.66
PHT In. Rel Hum.	7.96
PHT Out. Rel. Hum.	7.73
PHT Out. Abs. Hum.	5.92
Exhaust Temp.	5.08
PHT Out. Pres.	5.04
Product Temp.	3.99
PHT In. Pres.	3.80
Prod. & Filt.diff.Pres.	3.25
Average NIR coeff. *	1.86 *
SD(NIR coeff) *	1.19 *
Max(NIR coeff) *	8.72 *

* Each of the four models contains process parameters and 256 wavelength variables. From the four regression vectors (containing absolute coefficient values), we take the average vector, which now contains the average process parameters and 256 average regression coefficient values. The marked values in this table are the (NIR) averages, SD, and max of these 256 average values.

## Data Availability

Data are contained within the article.

## Supplementary Materials

The following supporting information can be downloaded at: https://www.mdpi.com/article/10.3390/pharmaceutics17060720/s1, Figure S1: (a) PC1-PC3 scores of the SNV NIR spectra. Purple: Batch ‘1214’. Red: Batch ‘1020’. All other batches are colored differently; (b) Selection of the optimal number of principal components. Natural logarithm of eigenvalues for PCA of the SNV-NIR spectra; Figure S2: Mahalanobis distance using the first three PCs of the SNVs of the NIR spectra for each of the 14 batches (instead on the first 5, as presented in Figure 4b); Figure S3: Combination PCA. Same analysis as in Figure 7, but instead of PC5 and PC7 scores, PC2 and PC3 scores are presented; Figure S4: Combination PCA. Same analysis as in Figure 7, but instead of PC5 and PC7 scores, PC2 and PC3 loading vectors are presented and in the color of the batch with the highest average value of the corresponding process parameter; Figure S5: Combination PCA. Same analysis as in Figure 7, but instead of PC5 and PC7 scores, PC5 and PC7 loading vectors are presented and in the color of the batch with the highest average value of corresponding process parameters; Figure S6: 3D plot of the PC2, PC5, and PC7 scores of the combination PCA; Figure S7: PC2 vs. PC5 plot of the combination PCA; Figure S8: SPP-PCA on scaled 22 process parameters (only, without NIR spectra). Red dots represent batch ‘1020’, blue dots represent ‘1214’, and brown dots ‘1213’. Note that PC5, PC7, and PC10 (2.5% var) had to be used to discriminate the ‘1020’ batch samples from other batches. Batch ‘1214’ could not be discriminated from other batches using any of the first 10 PCs; Figure S9: SPP-PCA on scaled 22 process parameters for 12 non-outlier batches. The figure clusters batches ‘0802’ and ‘0930’ from the rest. Batches 1212, 1213, 1229, and 1230 are not far from being separated from the other six batches; Figure S10: SPP-PCA. This PC2-PC3-PC4 plot separates batches ‘1212’, ‘1213’, ‘1229’, and ‘1230’ from ‘0920’, ‘1005’,’1011’, ‘1128’, ‘1129’, and ‘1203’ (for labels, see Figure S9); Figure S11: Airflow rate, inlet air temperature, atomization pressure, and pump speed for two outlier and two non-outlier batches. Table S1a: Process parameter average values for each batches containing samples with LOD values. Total of 14 batches containing 174 samples; Table S1b: Process parameter standard deviations for each batch containing samples with LOD values. A total of 14 batches containing 174 samples; Table S1c: Process parameter maximum values for each batch containing samples with LOD values. A total of 14 batches containing 174 samples; Table S1d: Process parameter minimum values for each batch containing samples with LOD values. A total of 14 batches containing 174 samples; Table S1e: Initial particle sizes for each batch. Table S1f: Total number of LOD measurements per batch. The sum gives 174; Table S2: Correlation analysis between 174 LOD samples; Table S3: Correlation between SNV-PC1 of the NIR spectra and process parameters batch-wise; Table S4: Correlation between SNV-PC1 of the NIR spectra and process parameters batch-wise; Table S5: Granule size statistics (Dv10, Dv25, Dv75, Dv90) for 12 non-outlier batches (for Dv50, see Table 1); Table S6: Comparison in the granule size external test RMSEP for MA 1 between the NIR-PLS model and merged-PLS (NIR+ 17-PP PLS). Extension of Table 2 and regarding Figure 8; Table S7: Comparison in the granule size internal test RMSEP for MA 1 between the NIR-PLS model and merged-PLS (NIR+ 17-PP PLS). See Figure 2 for the difference between the internal and external test sets; Table S8: Comparison in granule sizes of training set RMSECV for MA 1 between the NIR-PLS model and merged-PLS (NIR+ 17-PP PLS). See Figure 2 related to the first 90% of the data—training set (i.e., 81% of the data) for each separate batch (MA 1); Table S9: Comparison in granule sizes of the training set RMSEC for MA 1 between the NIR-PLS model and merged-PLS (NIR+ 17-PP PLS). See Figure 2 related to the first 90% of the data—training set (i.e., 81% of the data) for each separate batch (MA 1); Table S10: Comparison in granule sizes of training set RMSECV for MA 1 between the NIR-PLS model and merged-PLS (NIR+ 17-PP PLS) using block-CV instead of sequential-CV (in Table S8); Table S11: External test RMSEP statistic of MA 1 for NIR + pars: The same as Table S6, but NIR+ 18-PP PLS is here considered to include the spray rate as the 18th variable. 50%-l.s. represents MA 1-add, with included spray-rate process variables; Table S12: Comparison in granule size prediction accuracy for MA 2c, i.e., for four-batch prediction data between the NIR-PLS model and merged-PLS (NIR+ 17-PP PLS) (using eight other batches); Table S13: Comparison in the granule size external test RMSEP for MA 1 between the NIR-PLS model and merged-PLS for NIR+ 16-PP PLS—without runtime; Table S14: Comparison in granule size prediction accuracy for MA 2a, i.e., for three-batch prediction data between the NIR-PLS model and merged-PLS (NIR+ 16-PP PLS) (using nine other batches) omitting runtime in PLS modeling, which led to a somewhat weaker performance (as can be seen when compared to Table 3); Table S15: Comparison in granule size prediction accuracy for MA 2b, i.e., for one-batch prediction data between the NIR-PLS model and merged-PLS (NIR+ 16-PP PLS) (using eleven other batches) omitting runtime in PLS modeling, which led to a somewhat weaker performance (as can be seen when compared to Table 4). Average Dv50 CV error for the NIR spectra, CV = 71.95 μm; for NIR + 16 proc. parameters, CV = 43.62 μm; UVE-PLS code; Table S16: Granule size external test RMSEP for MA 1 of the Pars-only PLS model (17-pars) (result for comparison with Table S6); Table S17: Granule size internal test RMSEP for MA 1 of the Pars-only PLS model (17-PP) (result for comparison with Table S7); Table S18: Granule size RMSECV for MA 1 of the Pars-only PLS model (17-PP) (result for comparison with Table S8); Table S19: Granule size RMSEC for MA 1 of the Pars-only PLS model (17-PP) (result for comparison with Table S9); Table S20: MA 2a, RMSEP, and R2te for MA 2a for the Pars-only model. Results are comparable with Table 3 and are all repeated here for more clarity; Table S21: MA 2b, RMSEP, and R2te for MA 2b for the Pars-only model. Results are comparable with Table 4 and are all repeated here for more clarity; Table S22: MA 2c, RMSEP, and R2te for MA 2c for the Pars-only model. Results are comparable with Table S12 and are all repeated here for more clarity.

## Abbreviations

The following abbreviations are used in this manuscript:

BBE	Backward Batch Elimination
CQA	Critical Quality Attribute
FBG	Fluid Bed Granulation
LM	Lactose Monohydrate
LOD	Loss on Drying
MA	Modeling Approaches
MCC	Microcrystalline Cellulose
NIR	Near Infrared
PAT	Process Analytical Technology
PCA	Principal Component Analysis
PLS	Partial Least Square
PVP	Polyvinylpyrrolidone K90
RMSE	Root Mean Squared Error
RMSECV	Root Mean Squared Error of Prediction Cross-Validation
RMSEP	Root Mean Squared Error of Prediction
SFV	Spatial Filter Velocimetry
SNV	Standard Normal Variate
SPP	Scaled Process Parameters
UVE	Uninformative-Variable Elimination
NIR + pars	Merged NIR and process parameter model
Pars-only	Process parameter-only model
