# Supplementary material for: Merging Real-Time NIR and Process Parameter Measurements in a Fluidized Bed Granulation Process to Predict Particle Size
Source: Pharmaceutics. 2025 May 29;17(6):720. doi: 10.3390/pharmaceutics17060720 (PMC12196058; doi:10.3390/pharmaceutics17060720)
Supplement: Supplementary file 1 [file pharmaceutics-17-00720-s001.zip › pharmaceutics-3575236-supplementary.pdf]

pharmaceutics

MDPI

Supplementary information

Merging Real-Time NIR and Process Parameter Measurements in a Fluidized Bed Granulation Process to Predict Particle Size

Ozren Jovic[1,2], Marcus O'Mahony[1,3], Samuel Solomon[4], David Egan[1], Chris O'Callaghan [5], Caroline McCormack[5], Ian Jones[5], Patrick Cronin[4], Gavin Walker[1,4] and Rabah Mouras[1,*]

[1] Pharmaceutical Manufacturing Technology Centre, Bernal Institute, Department of Chemical Sciences, University of Limerick, V94 T9PX Limerick, Ireland ; ozren.jovic@universityofgalway.ie (O.J.); marcus.omahony@ul.ie (M.O.); david.egan@ul.ie (D.E.); gavin.walker@ul.ie (G.M.W.); rabah.mouras@ul.ie (R.M.)
[2] Dairy Processing Technology Centre, Bernal Institute, Department of Chemical Sciences, University of Limerick, V94 T9PX Limerick, Ireland; samuelsolomon2009@yahoo.ie (S.S.); patrick.cronin@imr.ie (P.C.)
[3] InnoGlobal Technology, Ravenscourt Campus, Dublin 18, D18 K599, Ireland; ocallaghanc@innoglobal.com (C.O.); mccormackc@innoglobal.com (C.M.); jonesi@innoglobal.com (I.J.); ocallaghanc@innoglobal.com (C.O.); mccormackc@innoglobal.com (C.M.); jonesi@innoglobal.com (I.J.)
[*] **Correspondence: rabah.mouras@ul.ie;**

Table S1a. Process parameter average values for each batch containing samples with LOD values. Total of 14 batches containing 174 samples.

	Moist	Airfl	Atom.p	Exh.p	Exh.Air.T	In.Air.T	Plen.Air.T	Prod.Fil.dP	Prod.T	Spray.r	PHT.HuA.In	PHT.p.In	PHT.HuR.in	PHT.T.In	PHT.HuA.O	PHT.p.O	PHT.HuR.O	PHT.T.O	Gran.S.10	Gran.S.50	Gran.S.90	Gran.Sp
20220802	5.941	24.684	0.953	-7.403	29.316	77.758	-0.343	7.060	30.379	11.157	13.847	1009.048	65.502	23.499	18.957	1009.180	17.988	55.438	132.754	274.990	482.936	1.247
20220920	5.289	25.294	1.124	-7.997	28.806	78.288	-0.331	7.666	30.065	8.172	8.752	1023.605	33.104	27.477	14.591	1023.819	10.222	62.436	160.975	307.081	507.756	1.117
20220930	7.598	34.867	1.267	-8.140	23.253	38.813	-0.428	7.712	21.813	7.726	9.090	997.167	37.960	25.702	12.514	996.919	21.155	45.362	240.746	420.309	678.482	1.006
20221005	7.719	33.500	1.333	-8.341	28.875	78.308	-0.482	7.859	30.525	17.621	7.119	1013.211	27.450	27.125	13.652	1012.987	10.288	60.739	191.834	345.948	593.362	1.153
20221011	9.322	34.600	1.360	-7.966	27.160	78.470	-0.488	7.478	28.130	16.206	7.599	1018.497	30.456	26.436	14.291	1018.213	11.843	58.402	205.200	360.122	602.819	1.087
20221020	12.252	36.000	1.700	-8.048	30.322	83.544	-0.502	7.546	31.889	31.942	9.621	995.762	35.349	28.005	17.464	995.941	15.555	56.517	186.369	337.158	583.846	1.203
20221128	7.609	29.364	0.991	-7.526	27.864	78.345	-0.382	7.145	28.073	12.929	7.032	1015.363	30.663	24.928	13.110	1014.679	9.459	61.421	166.795	341.902	604.441	1.236
20221129	7.417	29.273	1.055	-7.375	27.109	79.173	-0.395	6.980	27.873	12.962	6.544	1015.305	28.645	24.864	12.475	1014.620	8.863	61.815	172.741	347.972	609.705	1.219
20221203	8.303	31.444	0.878	-7.810	25.956	78.511	-0.384	7.426	25.922	16.016	5.589	1019.480	25.981	23.795	12.009	1018.909	9.149	60.392	177.732	379.174	670.082	1.280
20221212	8.670	31.222	1.333	-7.810	24.122	76.400	-0.247	7.563	25.200	15.805	3.601	1005.025	23.075	18.273	9.725	1004.269	7.554	59.948	199.598	370.149	638.542	1.162
20221213	4.743	18.700	1.410	-6.851	25.070	71.000	-0.029	6.822	26.550	12.460	4.129	1005.182	26.389	18.320	7.564	1004.966	6.611	56.994	116.510	235.906	380.770	1.148
20221214	6.893	32.267	1.200	-7.977	27.087	77.113	-0.285	7.693	29.460	8.722	3.794	1012.319	24.383	18.228	9.652	1012.159	9.279	54.867	207.619	430.588	707.408	1.185
20221229	9.048	26.462	1.192	-7.080	25.285	76.300	-0.319	6.761	25.354	10.704	5.720	992.669	29.852	23.241	11.028	991.919	8.578	61.607	184.113	376.294	693.913	1.313
20221230	7.880	24.571	1.286	-7.398	28.043	76.879	-0.334	7.064	28.336	9.800	6.400	989.414	30.218	23.527	12.104	988.630	9.526	59.166	173.275	339.435	617.016	1.292
MAX	12.3	36.0	1.7	-6.9	30.3	83.5	0.0	7.9	31.9	31.9	13.8	1023.6	65.5	28.0	19.0	1023.8	21.2	62.4	240.7	430.6	707.4	1.3
MIN	4.7	18.7	0.9	-8.3	23.3	38.8	-0.5	6.8	21.8	7.7	3.6	989.4	23.1	18.2	7.6	988.6	6.6	45.4	116.5	235.9	380.8	1.0
Batch max	1020	1020	1020	1213	1020	1020	1213	1005	1020	1020	802	920	802	1020	802	920	930	920	930	1214	1214	1229
Batch min	1213	1213	1203	1005	930	930	1020	1229	930	930	1212	1230	1212	1214	1213	1230	1213	930	1213	1213	1213	930

Table S1b. Process parameters standard deviations for each batch containing samples with LOD values. Total of 14 batches containing 174 samples.

	Moist	Airfl	Atom.p	Exh.p	Exh.Air.T	In.Air.T	Plen.Air.T	Prod.Fil.dP	Prod.T	Spray.r	PHT.HuA.In	PHT.p.In	PHT.HuR.in	PHT.T.In	PHT.HuA.O	PHT.p.O	PHT.HuR.O	PHT.T.O	Gran.S.10	Gran.S.50	Gran.S.90	Gran.Sp
20220802	2.856	7.789	0.382	0.941	1.799	3.463	0.164	0.887	2.596	8.249	0.217	0.055	1.343	0.202	1.732	0.137	2.927	4.389	34.179	61.234	159.681	0.235
20220920	2.991	7.051	0.412	1.010	3.374	3.353	0.117	0.986	5.852	9.497	0.113	0.138	0.331	0.243	2.111	0.328	2.215	2.335	30.018	43.834	111.550	0.204
20220930	4.548	8.806	0.258	1.447	4.277	16.557	0.161	1.369	3.501	8.974	0.117	0.224	0.977	0.237	1.518	0.354	8.586	12.969	67.590	133.852	259.284	0.203
20221005	4.963	11.017	0.078	1.414	2.760	3.892	0.132	1.371	6.805	10.373	0.156	0.822	0.637	0.179	2.989	0.831	3.040	4.624	67.035	124.974	231.501	0.246
20221011	5.359	9.477	0.097	1.019	1.322	3.572	0.188	0.871	4.083	12.815	0.245	0.230	1.100	0.122	1.926	0.238	2.286	2.024	64.873	100.682	203.618	0.185
20221020	6.368	13.370	0.000	1.720	2.258	4.283	0.193	1.615	7.097	12.881	0.046	0.272	0.426	0.147	2.603	0.249	1.961	3.269	100.253	168.132	293.464	0.145
20221128	3.981	6.772	0.327	0.466	2.111	3.946	0.108	0.431	3.412	9.575	0.062	0.311	0.375	0.102	1.512	0.309	1.354	1.923	41.976	86.071	221.522	0.245
20221129	3.891	7.171	0.353	0.949	1.807	2.732	0.135	0.900	3.483	11.031	0.069	0.096	0.238	0.060	1.645	0.144	1.505	1.936	46.592	102.061	225.176	0.222
20221203	3.530	10.273	0.233	0.843	1.515	2.586	0.178	0.709	1.960	6.529	0.045	0.056	0.340	0.108	2.112	0.063	2.316	2.817	58.094	133.433	250.436	0.193
20221212	5.112	6.852	0.158	0.534	1.618	4.981	0.104	0.489	4.079	17.143	0.020	0.079	0.126	0.074	1.539	0.122	1.647	2.235	47.892	88.026	189.913	0.159
20221213	2.572	2.541	0.145	0.749	2.819	1.614	0.213	0.769	4.711	19.100	0.029	0.149	0.548	0.247	1.585	0.130	1.273	3.787	31.381	51.008	75.505	0.213
20221214	5.086	12.759	0.254	1.343	5.349	4.429	0.208	1.229	7.636	7.613	0.060	0.237	0.710	0.235	2.625	0.213	2.798	3.130	89.145	189.607	285.076	0.236
20221229	4.121	6.565	0.253	0.462	1.278	4.713	0.147	0.401	1.502	9.967	1.731	0.750	0.797	0.183	3.635	0.816	0.964	1.216	42.638	94.808	227.992	0.236
20221230	4.553	6.465	0.257	1.128	3.363	3.599	0.162	1.183	4.727	12.404	0.044	0.120	0.552	0.207	1.843	0.084	0.918	2.291	57.486	109.596	231.099	0.250
Min	2.572	2.541	0.000	0.462	1.278	1.614	0.104	0.401	1.502	6.529	0.020	0.055	0.126	0.060	1.512	0.063	0.918	1.216	30.018	43.834	75.505	0.145
Max	6.368	13.370	0.412	1.720	5.349	16.557	0.213	1.615	7.636	19.100	1.731	0.822	1.343	0.247	3.635	0.831	8.586	12.969	100.253	189.607	293.464	0.250
Batch max	1020	1020	920	1020	1214	930	1213	1020	1214	1213	1229	1005	802	1213	1229	1005	930	930	1020	1214	1020	1230
Batch min	1213	1213	1020	1229	1229	1213	1212	1229	1229	1203	1212	802	1212	1229	1228	1203	1230	1229	920	920	1213	1020

pharmaceutics

MDPI

Table S1c. Process parameters maximum values for each batch containing samples with LOD values. Total of 14 batches containing 174 samples.

	Moist	Airfl	Atom.p	Exh.p	Exh.Air.T	In.Air.T	Plen.Air.T	Prod.Fil.dP	Prod.T	Spray.r	PHT.HuA.In	PHT.p.In	PHT.HuR.in	PHT.T.In	PHT.HuA.O	PHT.p.O	PHT.HuR.O	PHT.T.O	Gran.S.10	Gran.S.50	Gran.S.90	Gran.Sp
20220802	10.28	42.0	1.5	-5.71	32.8	82.1	-0.06	8.40	36.4	24.267	14.096	1009.2	67.155	23.862	21.267	1009.4	22.153	61.555	194.93	384.33	807.9	1.684
20220920	10.31	41.0	1.5	-6.93	39.1	81.8	-0.21	10.53	46.7	27.181	8.953	1023.8	33.832	27.836	17.563	1024.3	12.782	66.462	216.82	380.68	733.9	1.677
20220930	14.80	53.0	1.5	-6.47	32.8	76.5	-0.21	11.14	30.0	25.511	9.244	997.4	39.247	26.086	15.014	997.3	29.841	68.570	332.99	621.63	1095.9	1.229
20221005	14.43	58.0	1.5	-6.78	35.1	81.3	-0.21	10.38	46.6	34.160	7.469	1014.7	28.720	27.383	18.454	1014.4	17.145	64.685	314.12	620.75	1020.0	1.598
20221011	16.21	52.0	1.5	-6.32	30.1	83.2	-0.21	9.31	39.0	36.153	7.824	1018.7	31.502	26.640	16.821	1018.5	15.611	61.164	310.95	551.23	950.3	1.402
20221020	20.07	57.0	1.7	-5.10	35.0	88.0	-0.06	10.07	49.6	64.614	9.670	996.0	35.667	28.356	20.724	996.2	20.038	59.895	335.98	591.79	1029.1	1.381
20221128	13.18	42.0	1.5	-6.78	33.3	81.8	-0.21	7.78	35.6	30.587	7.077	1015.8	31.117	25.048	15.152	1015.1	11.901	63.834	235.06	471.13	956.2	1.692
20221129	12.76	44.0	1.5	-6.17	31.7	82.5	-0.21	8.85	36.5	28.100	6.683	1015.4	29.172	24.997	15.216	1014.8	11.378	64.226	276.17	581.24	1015.4	1.660
20221203	12.47	48.0	1.5	-6.78	29.7	81.1	-0.06	9.00	30.8	23.093	5.633	1019.6	26.497	23.917	14.887	1019.0	12.865	63.287	270.62	603.49	965.9	1.558
20221212	15.76	39.0	1.5	-6.78	26.9	81.6	-0.06	8.25	35.6	48.507	3.622	1005.2	23.284	18.438	11.654	1004.4	9.809	62.597	258.82	490.09	907.2	1.356
20221213	8.82	23.0	1.5	-5.86	30.0	72.8	0.55	8.70	36.0	55.863	4.194	1005.4	27.249	18.689	9.167	1005.2	8.207	61.258	157.70	307.68	529.5	1.699
20221214	14.13	48.0	1.5	-5.56	41.3	81.5	-0.06	10.23	46.5	18.533	3.885	1012.7	25.514	18.517	13.610	1012.5	14.982	58.951	363.30	728.52	1050.5	1.740
20221229	15.17	35.0	1.5	-6.47	29.0	82.1	-0.06	7.33	28.8	31.327	6.495	994.0	30.871	23.662	13.738	993.4	9.854	63.106	244.67	517.00	948.4	1.622
20221230	14.83	34.0	1.5	-5.86	35.1	82.0	-0.06	10.84	40.9	35.932	6.484	989.6	31.373	23.764	14.177	988.7	11.028	61.050	256.17	503.72	933.8	1.592
MAX	20.07	58.0	1.7	-5.10	41.3	88.0	0.55	11.14	49.6	64.614	14.096	1023.8	67.155	28.356	21.267	1024.3	29.841	68.570	363.30	728.52	1095.9	1.740
MIN	8.82	23.0	1.5	-6.93	26.9	72.8	-0.21	7.33	28.8	18.533	3.622	989.6	23.284	18.438	9.167	988.7	8.207	58.951	157.70	307.68	529.5	1.229
batch max	1020	1005	1020	1020	1214	1020	1213	930	1020	1020	802	920	802	1020	802	920	930	930	1214	1214	930	1214
batch min	1213	1213	802	920	1212	1213	920	1229	1229	1214	1212	1230	1212	1212	1213	1230	1213	1214	1213	1213	1213	930

Table S1d. Process parameters minimum values for each batch containing samples with LOD values. Total of 14 batches contain 174 samples.

	Moist	Airfl	Atom.p	Exh.p	Exh.Air.T	In.Air.T	Plen.Air.T	Prod.Fil.dP	Prod.T	Spray.r	PHT.HuA.In	PHT.p.In	PHT.HuR.in	PHT.T.In	PHT.HuA.O	PHT.p.O	PHT.HuR.O	PHT.T.O	Gran.S.10	Gran.S.50	Gran.S.90	Gran.Sp
20220802	1.87	11	0.70	-9.07	27.10	70.50	-0.67	5.65	28.50	0.00	13.39	1009.0	62.45	23.12	15.32	1008.9	12.19	46.30	63.01	141.90	253.24	0.92
20220920	1.37	12	0.70	-10.74	26.60	69.80	-0.52	6.72	26.40	-0.09	8.54	1023.3	32.64	27.08	10.33	1023.2	6.43	58.50	120.81	241.09	363.16	0.94
20220930	2.61	23	1.00	-11.66	19.50	26.70	-0.67	5.95	18.20	0.00	8.90	996.8	36.37	25.37	10.26	996.2	7.70	32.42	137.22	241.94	361.65	0.68
20221005	1.45	19	1.30	-11.05	26.60	68.80	-0.67	6.41	25.70	0.00	6.93	1012.4	26.31	26.80	7.91	1012.1	5.85	50.52	64.15	161.18	321.73	0.76
20221011	2.09	22	1.30	-9.98	26.10	70.10	-0.67	6.11	25.00	0.00	7.24	1018.2	28.83	26.25	11.24	1017.8	9.62	55.23	127.64	229.74	339.22	0.75
20221020	2.55	16	1.70	-10.74	28.00	73.60	-0.67	4.58	28.10	21.98	9.52	995.2	34.35	27.90	11.84	995.4	13.62	49.32	48.27	67.13	140.34	1.02
20221128	1.87	20	0.80	-8.15	26.10	67.40	-0.52	6.56	26.00	0.00	6.88	1014.9	29.90	24.75	10.16	1014.2	8.16	58.77	81.22	216.21	329.53	0.96
20221129	1.74	20	0.80	-9.37	25.70	73.30	-0.52	5.80	25.60	-0.13	6.47	1015.2	28.43	24.77	9.83	1014.3	6.96	58.80	99.61	222.54	338.12	0.90
20221203	2.86	16	0.80	-9.52	25.00	72.70	-0.67	6.57	24.50	0.00	5.51	1019.4	25.48	23.59	7.99	1018.8	6.56	55.39	69.57	190.00	308.85	0.97
20221212	1.71	19	1.20	-8.46	22.90	68.00	-0.37	6.57	22.40	0.00	3.56	1004.9	22.89	18.17	6.90	1004.0	4.91	57.08	122.90	223.10	334.16	0.95
20221213	1.62	15	1.20	-8.76	22.30	68.10	-0.21	5.80	22.80	0.00	4.10	1005.0	25.70	17.88	4.73	1004.8	4.14	48.75	62.38	125.72	275.95	0.93
20221214	1.04	15	1.00	-10.29	23.10	69.80	-0.67	5.50	22.80	0.00	3.70	1011.9	23.43	17.77	4.37	1011.6	5.37	49.54	63.87	148.72	279.30	0.94
20221229	2.77	15	1.00	-7.84	24.30	67.40	-0.52	6.25	23.50	0.00	0.00	991.7	28.50	22.99	0.00	990.8	7.18	58.38	86.95	213.75	328.14	0.88
20221230	1.85	14	1.00	-10.90	25.50	71.40	-0.67	5.65	25.40	0.00	6.36	989.2	29.74	23.10	7.28	988.5	7.70	52.74	63.03	144.62	293.27	0.84
MAX	2.86	23.0	1.70	-7.84	28.00	73.60	-0.21	6.72	28.50	21.98	13.39	1023.3	62.45	27.90	15.32	1023.2	13.624	58.798	137.22	241.94	363.2	1.024
MIN	1.04	11.0	0.70	-11.66	19.50	26.70	-0.67	4.58	18.20	-0.13	0.00	989.2	22.89	17.77	0.00	988.5	4.141	32.422	48.27	67.13	140.3	0.684
batch max	1203	930	1020	1229	1020	1020	1213	920	802	1020	802	920	802	1020	802	920	1020	1129	930	930	920	1020
batch min	1214	802	802	930	930	930	802	1020	930	1129	1229	1230	1212	1214	1229	1230	1129	930	1020	1020	1020	930

Table S1e. Initial particle sizes for each batch.

Batch	Dv10/µm	Dv25/µm	Dv50/µm	Dv75/µm	Dv90/µm
0802	81.11	140.20	197.94	264.34	310.47
0920	66.97	89.78	158.6	220.55	266.25
0930	67	91.54	172.18	236.34	297.7
1005	66.07	84.13	154.82	223.8	286.17
1011	70.4	130.78	181.39	243.02	316.18
1020	76.81	144.59	202.08	247.75	320.42
1128	42.14	50.32	62.14	100.34	122.66
1129	65.07	80.16	145.68	183.39	236.13
1203	65.76	87.46	160.91	232.82	293.42
1212	45.18	51.65	59.31	72.07	126.36
1213	66.66	87.8	152.27	214.45	247.62
1214	66.42	85.18	149.7	202.23	236.98
1229	63.63	74.97	144.01	212.66	268.8
1230	65.28	81.25	145.06	202.43	239.48

pharmaceutics

MDPI

Table S1f. Total number of LOD measurements per batch. The sum gives 174.

Batch code	0802	0920	0930	1005	1011	1020	1128	1129	1203	1212	1213	1214	1229	1230
No. LOD measur.	19	17	15	12	10	9	11	11	9	9	10	15	13	14

Moist: Moisture level, which is LOD in % w/w (mass fraction of water in wet product).
Airfl: Airflow rate in cubic meters per hour (m3/h).
Atom.p: Atomization pressure in bar.
Exh.p: Exhaust (Air) pressure in mbar.
Exh.Air.T.: Exhaust Air Temperature in °C.
In.Air.T: Inlet Air Temperature in °C
Plen.Air.T: Plenum air pressure in mbar.
Prod.Fil.dP: Product and filter delta P in mbar.
Prod.T: Product temperature in °C.
Spray.r: Spray rate average in g/min.
PHT.HuA.In: Pressure-Humidity-Temperature (PHT) Sensor measuring inlet absolute humidity in g/m3 (grams per cubic meter).
PHT.p.In. PHT Sensor measuring inlet pressure in mbar.
PHT.HuR.In. PHT sensor measuring relative humidity inlet in % of partial pressure of water vapor and saturation vapor pressure.
PHT.T.In. - PHT Sensor measuring inlet temperature in °C.
PHT.HuA.O - PHT sensor measuring absolute humidity outlet in g/m3.
PHT.p.O - PHT sensor measuring outlet pressure in mbar.
PHT.HuR.O. - PHT sensor measuring relative humidity outlet in %.
PHT.T.O. - PHT sensor measuring outlet temperature in °C.
Gran.S.10. - Granule Size Dv10 (μm).
Gran.S.50. - Granule Size Dv50 (μm).
Gran.S.90. - Granule Size Dv90 (μm).
Gran.Sp. - Granule Span.
'MAX' - represents maximum column value. 'MIN' - minimum column value. 'Batch max' - the batch (i.e. last four defining numerals) with the maximum column value. 'Batch min' - the batch (i.e. last four defining numerals) with the minimum column value.
Additional comment for clarification: For example, all 19 LOD values for batch '0802' in % w/w are: 1.87, 2.23, 2.53, 2.7, 4.03, 4.74, 5.9, 7.07, 7.78, 7.33, 9.51, 10.23, 10.28, 9.19, 8.47, 7.12, 5.29, 3.5, 3.11. Their average value is 5.941 % (Table S1a, first row, first column), standard deviation is 2.856 (Table S1b), maximum value 10.28 (Table S1c), minimum value 1.87 (Table S1d).

Table S2. Correlation analysis between 174 LOD samples.

	Moist	AirFl	Atom.p.	Exh.p	Exh.Air.T	In.Air.T	Plen.Air.p	Prod.Fil.dP	Prod.T	Spray.r	PHT.HuA.In	PHT.p.In	PHT.HuR.in	PHT.T.In	PHT.HuA.O	PHT.p.O	PHT.HuR.O	PHT.T.O	Gran.S.10	Gran.S.50	Gran.S.90	Gran.Sp
Moist	1.000	0.665	-0.084	-0.389	-0.395	0.149	-0.512	0.323	-0.442	0.336	-0.048	-0.157	-0.095	0.107	0.369	-0.158	0.128	0.113	0.576	0.625	0.716	0.442
AirFl	0.665	1.000	0.025	-0.674	-0.230	0.010	-0.709	0.591	-0.231	0.113	-0.030	0.028	-0.117	0.162	0.378	0.030	0.337	-0.146	0.861	0.858	0.822	0.134
Atom.p.	-0.084	0.025	1.000	0.114	-0.092	-0.067	0.033	-0.116	0.160	-0.397	-0.164	-0.264	-0.227	0.040	-0.203	-0.261	0.024	-0.179	0.167	0.088	0.081	-0.026
Exh.p	-0.389	-0.674	0.114	1.000	-0.063	0.045	0.443	-0.987	0.031	-0.145	-0.035	-0.118	0.046	-0.156	-0.217	-0.122	-0.207	0.133	-0.546	-0.558	-0.532	-0.183
Exh.Air.T	-0.395	-0.230	-0.092	-0.063	1.000	0.398	0.008	0.069	0.814	0.155	0.272	0.168	0.212	0.237	0.116	0.174	-0.210	0.244	-0.386	-0.373	-0.379	-0.053
In.Air.T	0.149	0.010	-0.067	0.045	0.398	1.000	-0.083	-0.063	0.363	0.166	-0.087	0.295	-0.076	-0.021	0.257	0.290	-0.556	0.756	-0.148	-0.051	0.034	0.281
Plen.Air.p	-0.512	-0.709	0.033	0.443	0.008	-0.083	1.000	-0.293	0.113	-0.125	-0.214	-0.044	-0.076	-0.447	-0.498	-0.046	-0.328	0.004	-0.557	-0.559	-0.562	-0.155
Prod.Fil.dP	0.323	0.591	-0.116	-0.987	0.069	-0.063	-0.293	1.000	-0.013	0.132	-0.001	0.118	-0.063	0.086	0.142	0.122	0.161	-0.142	0.482	0.495	0.466	0.167
Prod.T	-0.442	-0.231	0.160	0.031	0.814	0.363	0.113	-0.013	1.000	-0.132	0.149	0.183	0.118	0.108	-0.045	0.186	-0.195	0.106	-0.364	-0.389	-0.398	-0.066
Spray.r	0.336	0.113	-0.397	-0.145	0.155	0.166	-0.125	0.132	-0.132	1.000	0.023	-0.033	-0.043	0.110	0.182	-0.029	-0.081	0.203	-0.073	-0.066	-0.063	-0.017
PHT.HuA.In	-0.048	-0.030	-0.164	-0.035	0.272	-0.087	-0.214	-0.001	0.149	0.023	1.000	0.045	0.915	0.529	0.794	0.064	0.630	-0.192	-0.132	-0.175	-0.161	-0.037
PHT.p.In	-0.157	0.028	-0.264	-0.118	0.168	0.295	-0.044	0.118	0.183	-0.033	0.045	1.000	-0.048	0.170	0.122	0.999	-0.169	0.255	-0.092	-0.065	-0.098	-0.095
PHT.HuR.in	-0.095	-0.117	-0.227	0.046	0.212	-0.076	-0.076	-0.063	0.118	-0.043	0.915	-0.048	1.000	0.196	0.657	-0.030	0.598	-0.243	-0.198	-0.197	-0.170	0.034
PHT.T.In	0.107	0.162	0.040	-0.156	0.237	-0.021	-0.447	0.086	0.108	0.110	0.529	0.170	0.196	1.000	0.522	0.177	0.299	0.093	0.079	-0.017	-0.005	-0.076
PHT.HuA.O	0.369	0.378	-0.203	-0.217	0.116	0.257	-0.498	0.142	-0.045	0.182	0.794	0.122	0.657	0.522	1.000	0.137	0.488	0.074	0.198	0.201	0.226	0.101
PHT.p.O	-0.158	0.030	-0.261	-0.122	0.174	0.290	-0.046	0.122	0.186	-0.029	0.064	0.999	-0.030	0.177	0.137	1.000	-0.151	0.243	-0.094	-0.068	-0.103	-0.098
PHT.HuR.O	0.128	0.337	0.024	-0.207	-0.210	-0.556	-0.328	0.161	-0.195	-0.081	0.630	-0.169	0.598	0.299	0.488	-0.151	1.000	-0.791	0.317	0.237	0.178	-0.140
PHT.T.O	0.113	-0.146	-0.179	0.133	0.244	0.756	0.004	-0.142	0.106	0.203	-0.192	0.255	-0.243	0.093	0.074	0.243	-0.791	1.000	-0.202	-0.129	-0.042	0.189
Gran.S.10	0.576	0.861	0.167	-0.546	-0.386	-0.148	-0.557	0.482	-0.364	-0.073	-0.132	-0.092	-0.198	0.079	0.198	-0.094	0.317	-0.202	1.000	0.939	0.859	-0.036
Gran.S.50	0.625	0.858	0.088	-0.558	-0.373	-0.051	-0.559	0.495	-0.389	-0.066	-0.175	-0.065	-0.197	-0.017	0.201	-0.068	0.237	-0.129	0.939	1.000	0.953	0.171
Gran.S.90	0.716	0.822	0.081	-0.532	-0.379	0.034	-0.562	0.466	-0.398	-0.063	-0.161	-0.098	-0.170	-0.005	0.226	-0.103	0.178	-0.042	0.859	0.953	1.000	0.436
Gran.Sp	0.442	0.134	-0.026	-0.183	-0.053	0.281	-0.155	0.167	-0.066	-0.017	-0.037	-0.095	0.034	-0.076	0.101	-0.098	-0.140	0.189	-0.036	0.171	0.436	1.000
	0.8>Cor>0.6			0.9>Cor>0.8			Cor>0.9															

pharmaceutics

MDPI

Table S3. Correlation between SNV-PC1 of NIR spectra and process parameters - batch-wise

	df (Ccor)	runtime	Moist	Airfl	Atom.p	Exh.p	Exh.Air.T	In.Air.T	Plen.Air.T	Prod.Fil.dP	Prod.T	Spray.r	PHT.HuA.In	PHT.p.In	PHT.HuR.in	PHT.T.In	PHT.HuA.O	PHT.p.O	PHT.HuR.O	PHT.T.O	Gran.S.10	Gran.S.50	Gran.S.90	Gran.Sp
20220802	17 (0.575)	-0.083	-0.371	-0.241	0.092	0.212	0.134	-0.229	0.100	-0.207	0.088	-0.345	-0.198	0.011	-0.085	-0.100	-0.131	0.024	0.042	-0.064	-0.180	-0.283	-0.333	-0.395
20220920	15 (0.606)	-0.037	-0.374	-0.605	0.268	0.684	-0.070	-0.523	0.236	-0.673	-0.073	-0.507	-0.010	0.117	0.140	-0.110	-0.241	-0.004	-0.139	-0.050	-0.404	-0.384	-0.328	-0.123
20220930	13 (0.641)	0.182	-0.717	-0.445	-0.035	0.345	0.223	0.242	-0.175	-0.386	0.421	0.068	0.064	-0.038	0.135	-0.191	-0.103	-0.095	0.039	0.051	-0.524	-0.607	-0.644	-0.639
20221005	10 (0.708)	-0.535	-0.496	-0.529	-0.189	0.229	0.375	-0.700	0.391	-0.198	0.116	0.185	-0.278	-0.531	0.036	-0.699	-0.513	-0.539	-0.602	0.294	-0.624	-0.539	-0.556	-0.038
20221011	8 (0.765)	-0.298	-0.736	-0.716	0.108	0.659	0.433	-0.599	0.780	-0.602	0.222	-0.012	-0.462	0.078	-0.401	-0.099	-0.683	-0.022	-0.553	0.107	-0.553	-0.649	-0.668	-0.439
20221020	7 (0.798)	-0.618	-0.680	-0.357	NA	-0.103	0.800	-0.658	0.408	0.159	-0.344	0.041	-0.453	0.605	-0.389	0.359	-0.465	0.554	0.043	-0.564	-0.046	0.003	-0.061	-0.389
20221128	9 (0.735)	-0.409	-0.562	-0.745	-0.287	0.651	0.501	-0.411	0.221	-0.649	0.304	0.401	0.051	-0.479	0.256	-0.466	-0.623	-0.460	-0.546	0.026	-0.445	-0.634	-0.692	-0.712
20221129	9 (0.735)	-0.388	-0.736	-0.873	-0.076	0.686	0.581	-0.483	0.322	-0.675	0.435	0.095	-0.207	-0.346	-0.178	-0.192	-0.821	-0.429	-0.817	0.300	-0.760	-0.885	-0.937	-0.814
20221203	7 (0.798)	-0.338	-0.543	-0.376	0.079	0.510	0.605	-0.538	0.667	-0.438	0.540	0.082	0.112	0.540	0.254	-0.387	-0.452	0.698	-0.240	-0.193	-0.410	-0.385	-0.379	-0.116
20221212	7 (0.798)	-0.655	-0.318	-0.816	-0.572	0.551	0.317	-0.831	0.439	-0.508	-0.095	0.377	-0.619	-0.015	-0.468	-0.205	-0.754	-0.391	-0.807	0.639	-0.758	-0.808	-0.789	-0.571
20221213	8 (0.765)	-0.653	0.037	-0.065	-0.237	-0.251	0.441	-0.627	0.259	0.316	0.082	0.328	0.639	-0.317	0.638	-0.600	-0.093	-0.134	0.355	-0.620	-0.078	-0.200	-0.126	0.219
20221214	13 (0.641)	-0.949	-0.074	-0.793	-0.443	0.315	-0.024	-0.922	0.704	-0.225	-0.237	0.398	0.931	-0.833	0.963	-0.948	-0.586	-0.671	-0.676	0.217	-0.817	-0.757	-0.750	0.240
20221229	11 (0.684)	-0.733	-0.564	-0.799	-0.503	0.807	0.389	-0.866	0.594	-0.712	-0.022	0.465	0.189	0.730	-0.765	-0.594	-0.087	0.736	-0.907	-0.163	-0.729	-0.847	-0.845	-0.680
20221230	12 (0.661)	-0.443	-0.396	-0.550	-0.341	-0.242	0.531	-0.493	0.463	0.294	0.550	0.308	0.572	-0.334	0.503	-0.451	-0.499	-0.296	-0.494	-0.355	-0.412	-0.511	-0.609	-0.465

Table S4. Correlation between SNV-PC1 of NIR spectra and process parameters - batch-wise

	df (Ccor)	runtime	Moist	Airfl	Atom.p	Exh.p	Exh.Air.T	In.Air.T	Plen.Air.T	Prod.Fil.dP	Prod.T	Spray.r	PHT.HuA.In	PHT.p.In	PHT.HuR.in	PHT.T.In	PHT.HuA.O	PHT.p.O	PHT.HuR.O	PHT.T.O	Gran.S.10	Gran.S.50	Gran.S.90	Gran.Sp
20220802	17 (0.575)	-0.270	-0.636	-0.451	-0.013	0.160	0.370	-0.537	0.284	-0.117	0.417	-0.222	-0.388	0.235	-0.114	-0.309	-0.414	0.113	0.215	-0.324	-0.471	-0.581	-0.580	-0.471
20220920	15 (0.606)	0.256	-0.719	-0.532	0.409	0.461	0.339	-0.519	0.310	-0.436	0.364	-0.562	0.284	-0.259	0.210	0.125	-0.612	-0.294	-0.502	0.280	-0.127	-0.313	-0.443	-0.485
20220930	13 (0.641)	0.251	-0.598	-0.486	0.089	0.373	0.157	0.171	-0.117	-0.408	0.379	-0.034	0.098	0.051	0.177	-0.244	-0.220	-0.004	0.114	-0.040	-0.468	-0.510	-0.564	-0.634
20221005	10 (0.708)	-0.371	-0.658	-0.622	0.014	0.225	0.631	-0.871	0.662	-0.168	0.414	-0.013	-0.438	-0.356	-0.134	-0.662	-0.725	-0.375	-0.604	0.059	-0.687	-0.650	-0.659	-0.035
20221011	8 (0.765)	-0.221	-0.721	-0.668	0.095	0.669	0.677	-0.670	0.828	-0.604	0.368	0.005	-0.369	0.013	-0.258	-0.403	-0.680	-0.066	-0.476	-0.068	-0.463	-0.605	-0.647	-0.559
20221020	7 (0.798)	0.001	-0.337	-0.492	NA	0.615	0.394	-0.374	0.325	-0.616	0.493	0.382	-0.458	-0.016	-0.275	0.176	-0.400	-0.055	0.058	-0.487	-0.629	-0.646	-0.699	-0.184
20221128	9 (0.735)	-0.423	-0.678	-0.733	-0.215	0.597	0.783	-0.722	0.147	-0.609	0.642	0.261	-0.031	-0.500	0.260	-0.596	-0.784	-0.510	-0.604	-0.130	-0.527	-0.613	-0.615	-0.561
20221129	9 (0.735)	-0.192	-0.807	-0.744	0.074	0.590	0.716	-0.614	0.235	-0.587	0.695	-0.069	0.014	-0.149	0.114	-0.230	-0.816	-0.191	-0.701	0.077	-0.687	-0.772	-0.838	-0.800
20221203	7 (0.798)	-0.387	-0.577	-0.419	0.037	0.526	0.729	-0.664	0.644	-0.464	0.672	0.104	0.159	0.586	0.317	-0.459	-0.522	0.735	-0.278	-0.235	-0.483	-0.421	-0.415	-0.095
20221212	7 (0.798)	-0.538	-0.492	-0.793	-0.459	0.404	0.593	-0.793	0.383	-0.359	0.175	0.193	-0.600	-0.017	-0.394	-0.265	-0.830	-0.323	-0.797	0.501	-0.718	-0.800	-0.806	-0.703
20221213	8 (0.765)	-0.379	-0.340	-0.383	-0.042	-0.299	0.399	-0.723	0.322	0.380	0.321	0.079	0.472	-0.011	0.423	-0.372	-0.368	0.137	-0.044	-0.471	-0.372	-0.475	-0.446	0.268
20221214	13 (0.641)	0.794	-0.597	0.467	0.842	-0.301	0.599	0.497	-0.489	0.246	0.823	-0.840	-0.820	0.465	-0.753	0.650	-0.079	0.187	0.220	-0.651	0.435	0.324	0.207	-0.436
20221229	11 (0.684)	-0.800	-0.493	-0.796	-0.582	0.702	0.545	-0.894	0.530	-0.615	0.118	0.501	0.201	0.807	-0.822	-0.682	-0.088	0.810	-0.921	-0.277	-0.730	-0.816	-0.829	-0.697
20221230	12 (0.661)	-0.512	-0.579	-0.686	-0.094	-0.565	0.720	-0.572	0.599	0.620	0.876	0.068	0.741	-0.246	0.695	-0.634	-0.800	-0.286	-0.688	-0.704	-0.651	-0.689	-0.696	-0.150

(a)

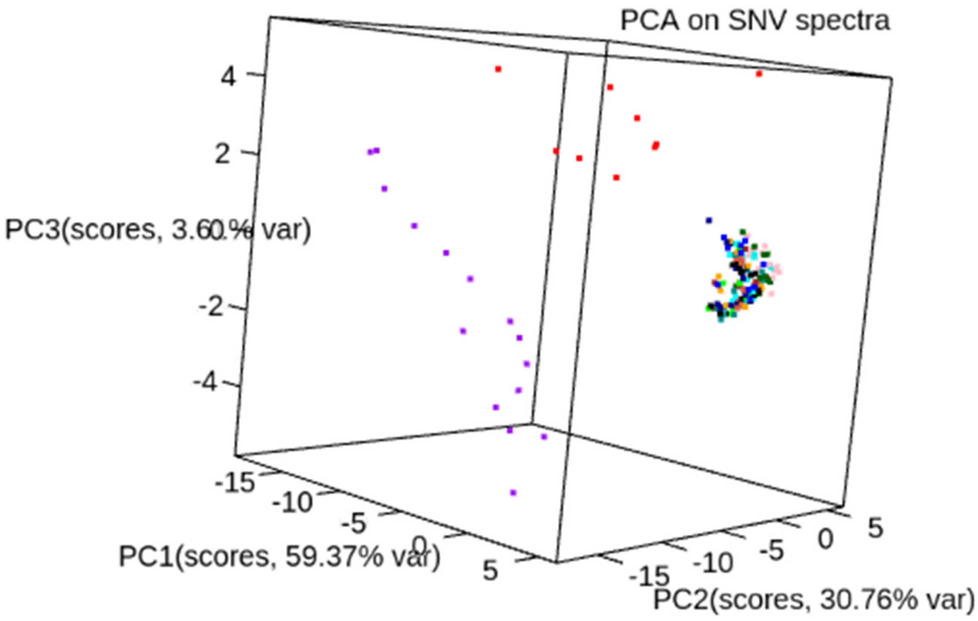

(b)

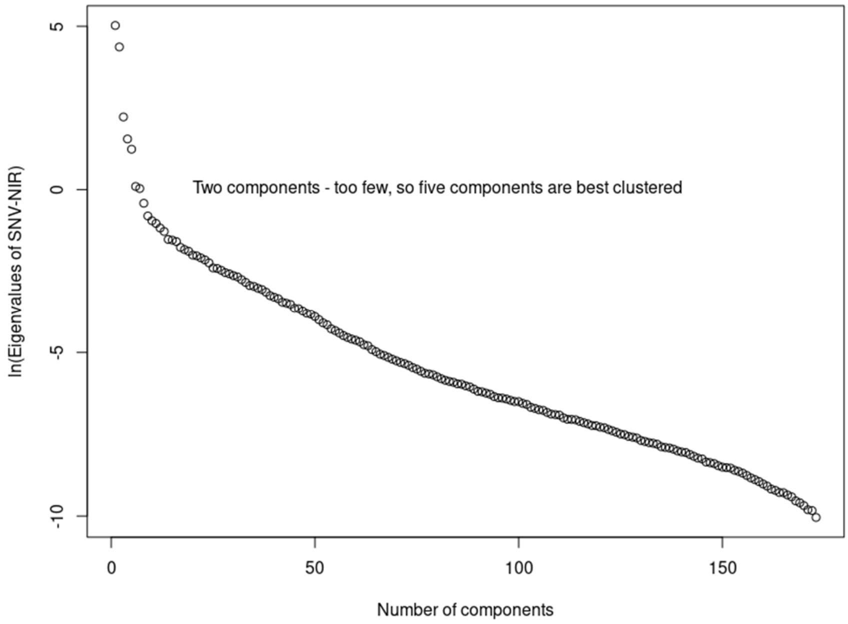

Figure S1. (a) PC1-PC3 scores of SNV NIR spectra. Purple: Batch '1214'. Red: Batch '1020'. All other batches are colored differently. (b) Selection of optimal number of principal components. Natural logarithm of Eigenvalues for PCA on SNV-NIR spectra. Two components would be in

pharmaceutics MDPI

severe conflict with Kaiser rule as components 3-5 are well above the margin of 0 (ln(1)=0, i.e. Value of 0 on this graph is the Kaiser margin). Five components wouldn't be that much and are best clustered. Seven components would still strictly follow Kaiser rule (but borderline), however as can be seen they are worse clustered than five components. Since it is already known that with Kaiser rule too many components might be selected, the decision for later Mahalanobis metric is to use the first five components.

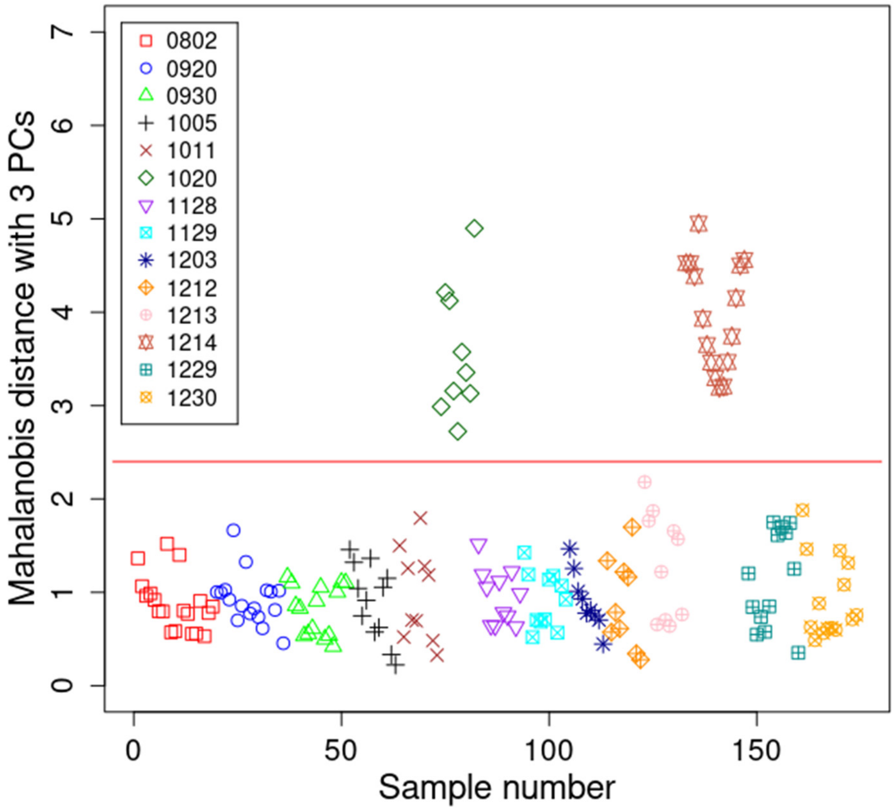

Figure S2. Mahalanobis distance using first three PC-s of SNV on NIR spectra for each of 14 batches (instead on first five as presented in Figure 4b).

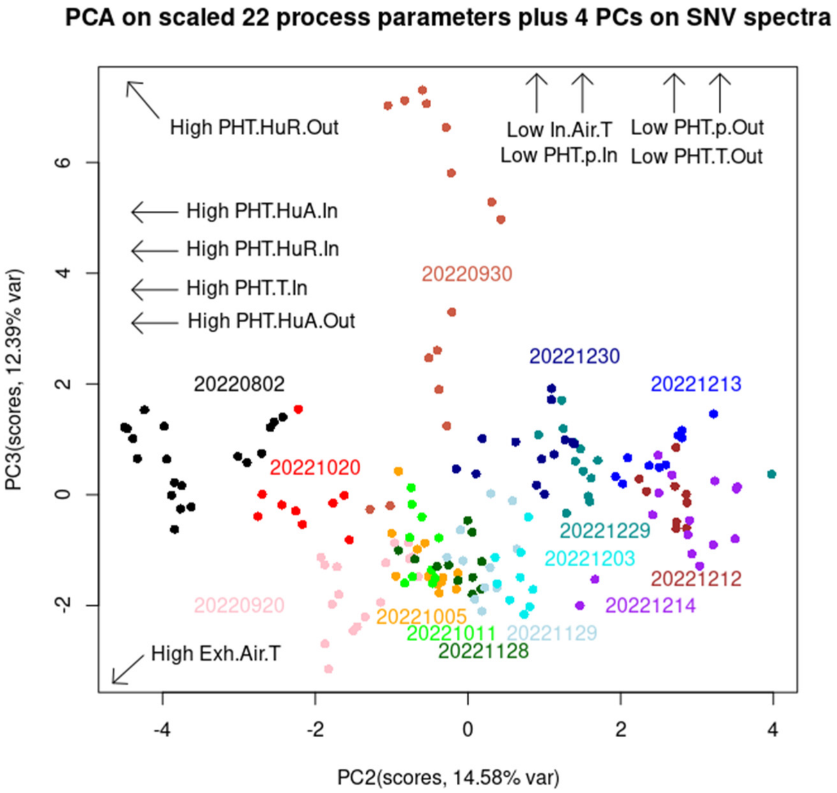

Figure S3. Combination PCA. Same analysis as in Figure 7, but instead PC5 and PC7 scores, presented are PC2 and PC3 scores.

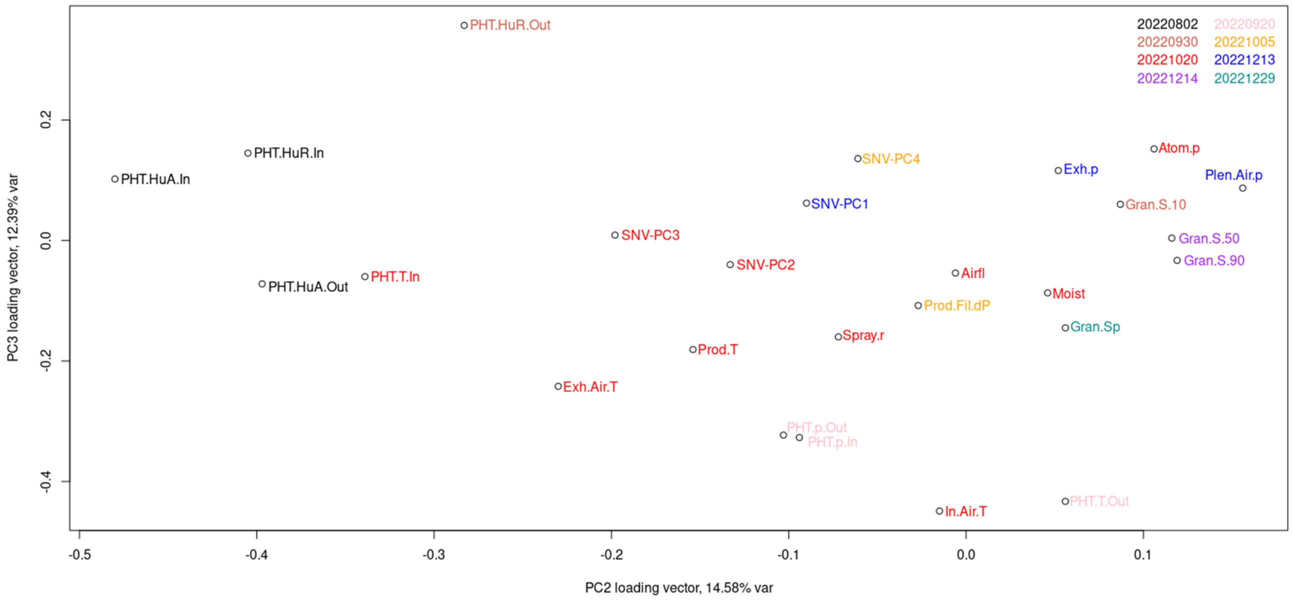

Figure S4. Combination PCA. Same analysis as in Figure 7, but instead PC5 and PC7 scores, presented are PC2 and PC3 loading vectors, and in color of the batch with the highest average value of corresponding process parameter.

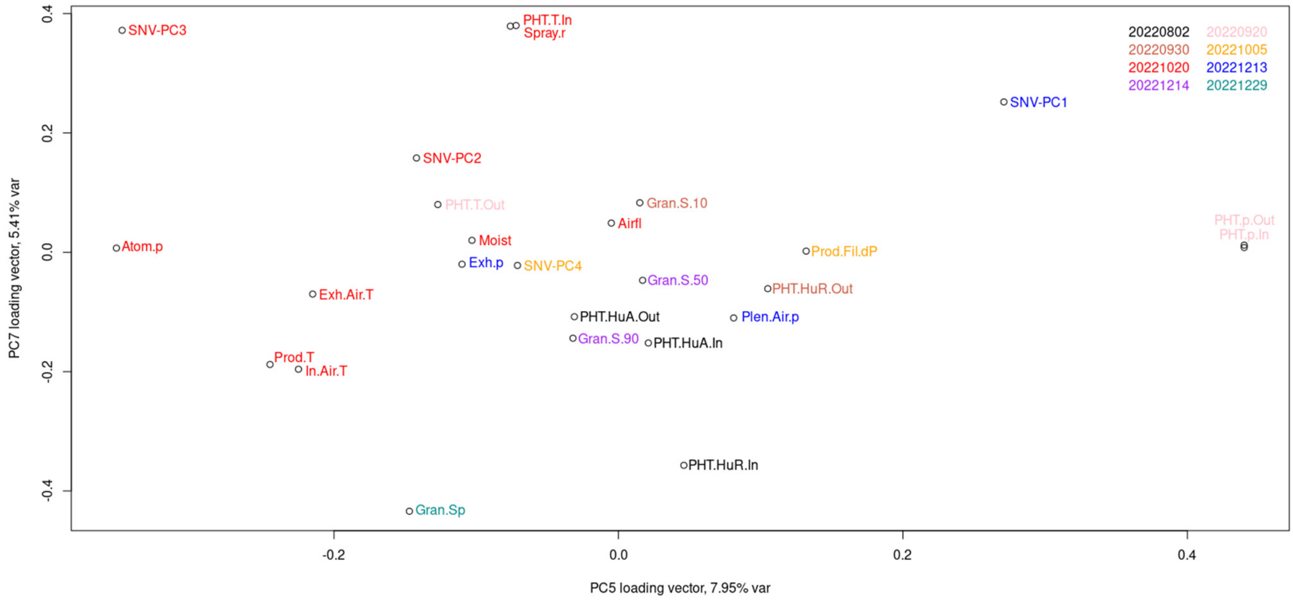

Figure S5. Combination PCA. Same analysis as in Figure 7, but instead PC5 and PC7 scores, presented are PC5 and PC7 loading vectors, and in color of the batch with the highest average value of corresponding process parameter.

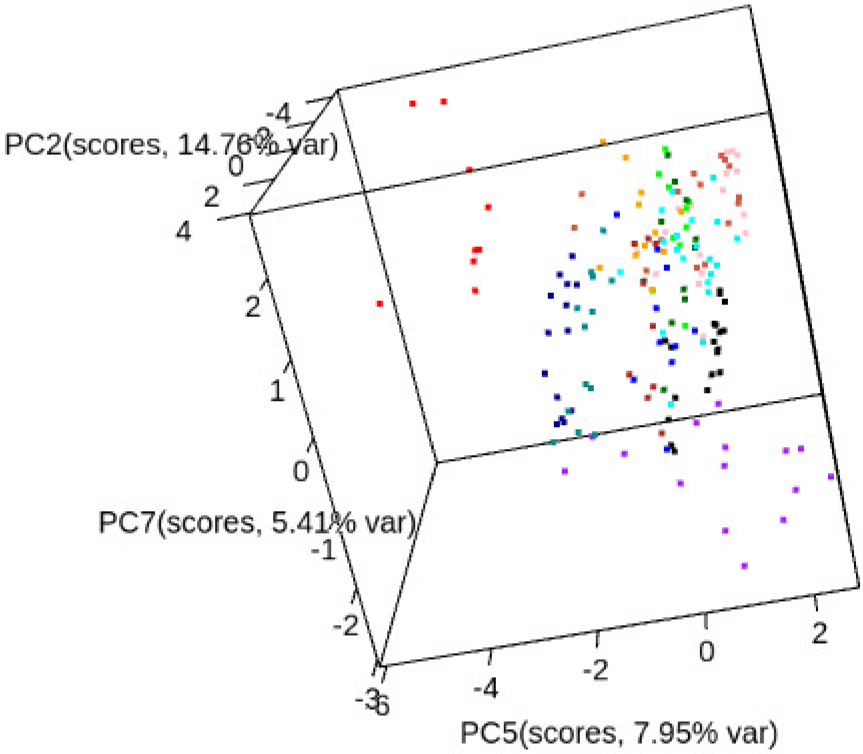

Figure S6. 3D plot of the PC2, PC5 and PC7 scores of the Combination PCA.

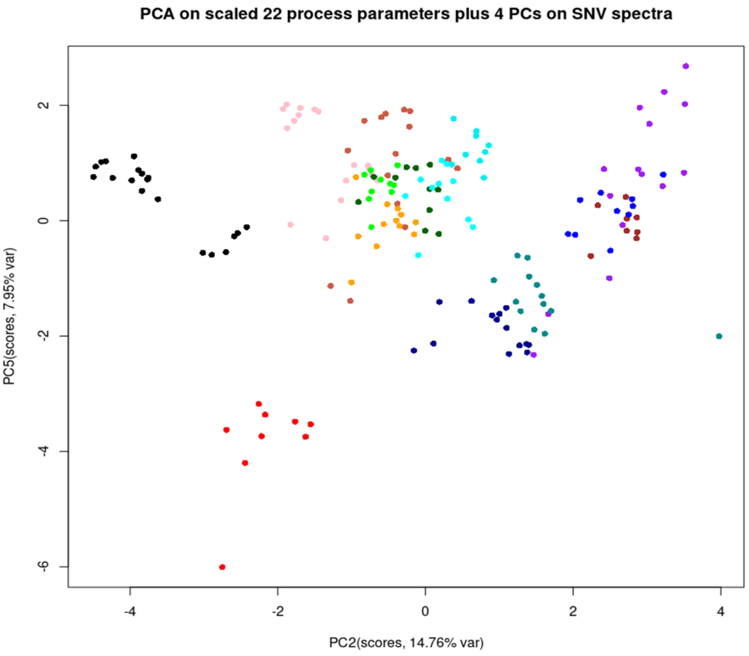

Figure S7. PC2 vs. PC5 plot of the Combination PCA.

pharmaceutics

MDPI

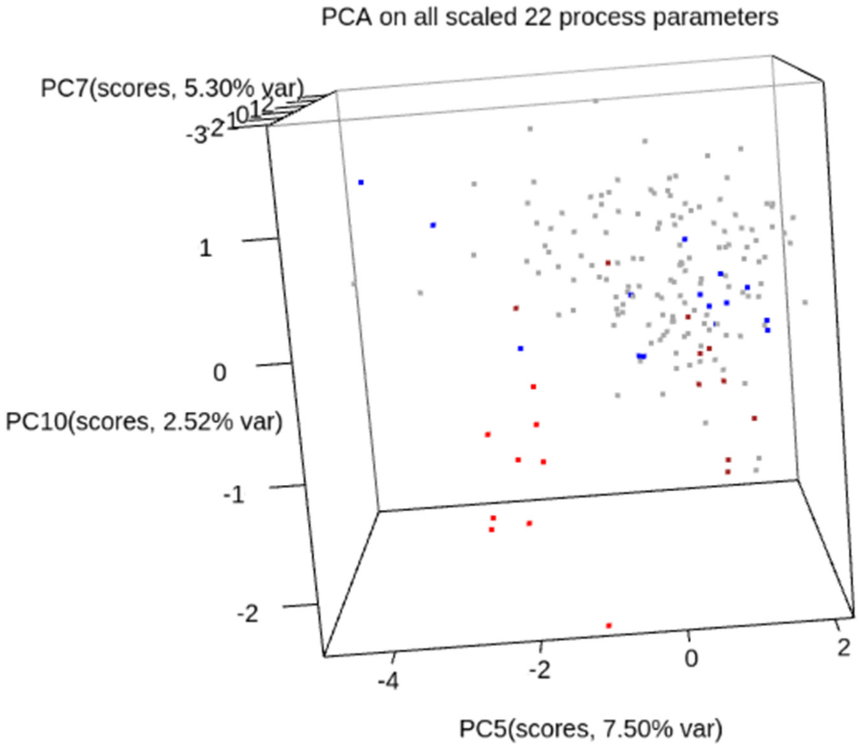

Figure S8. SPP-PCA on scaled 22 process parameters (only, so, without NIR spectra). Red dots represent batch '1020'. Blue dots represent '1214' and brown dots '1213' batch. Note that PC5, PC7 and PC10 (2.5% var) had to be used to discriminated all '1020' batch samples from other batches. Batch '1214' could not be discriminated from other batches by use of any first 10 PCs.

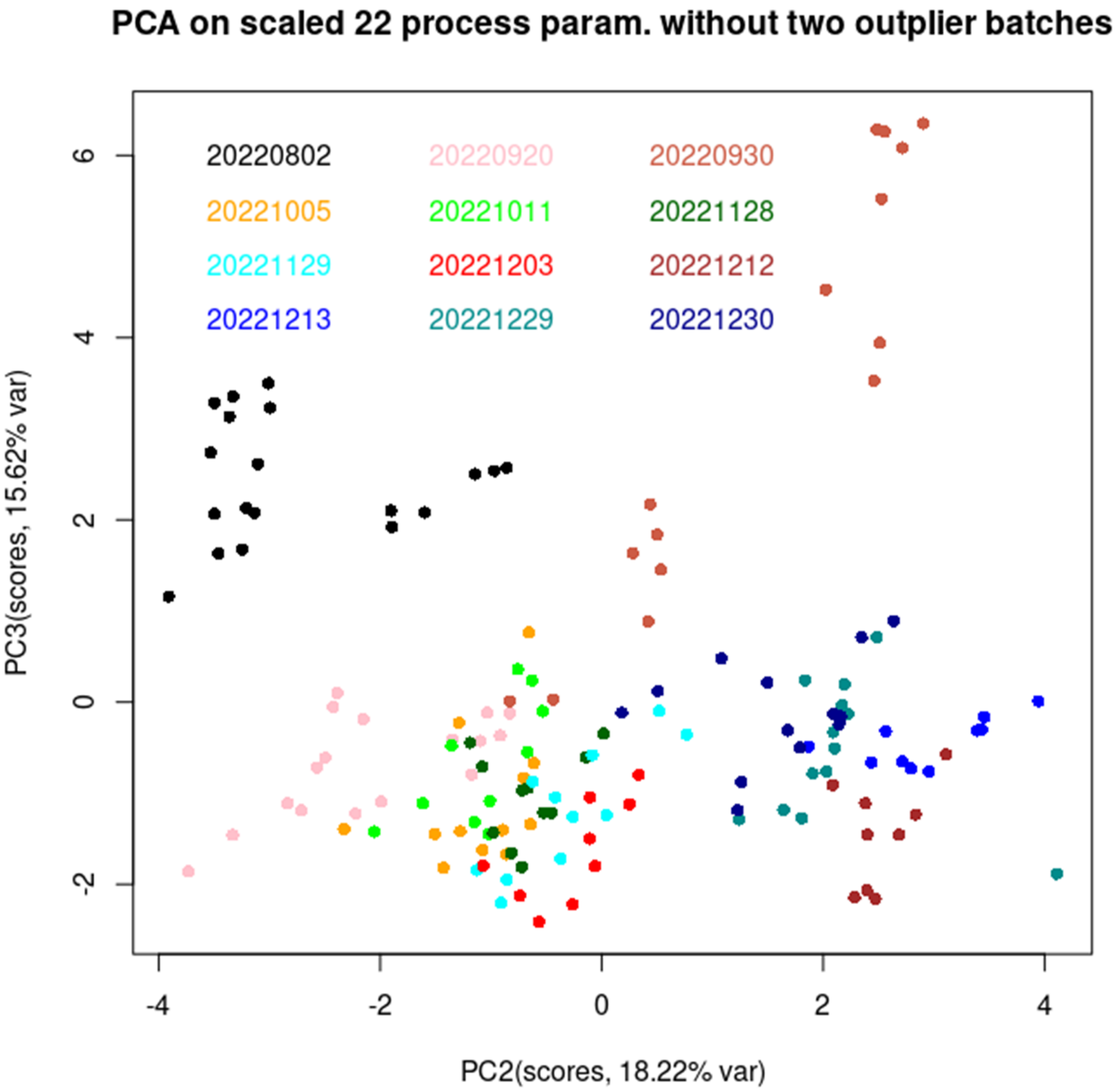

Figure S9. SPP-PCA on scaled 22 process parameters for 12 non-outlier batches. The figure clusters batches '0802' and '0930' from the rest. Batches 1212, 1213, 1229 and 1230 are not far from being separated from the other six batches.

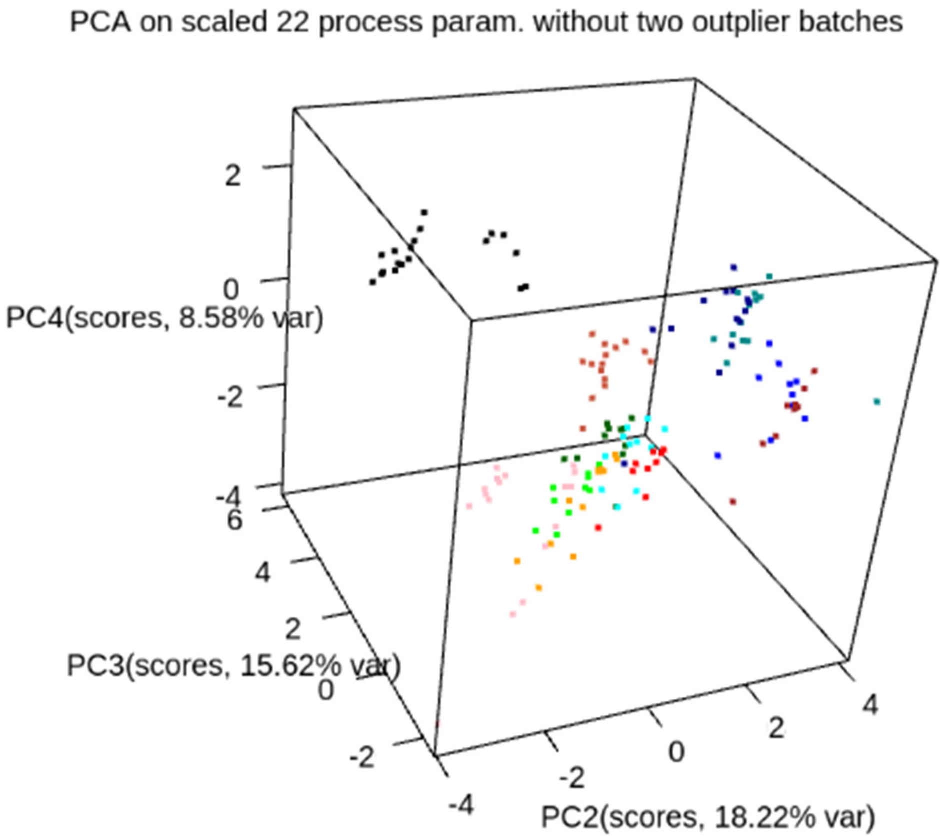

Figure S10. SPP-PCA. This PC2-PC3-PC4 plot separates batches '1212', '1213', '1229', and '1230' from '0920','1005','1011','1128','1129', and '1203' (for labels see Figure S9).

pharmaceutics

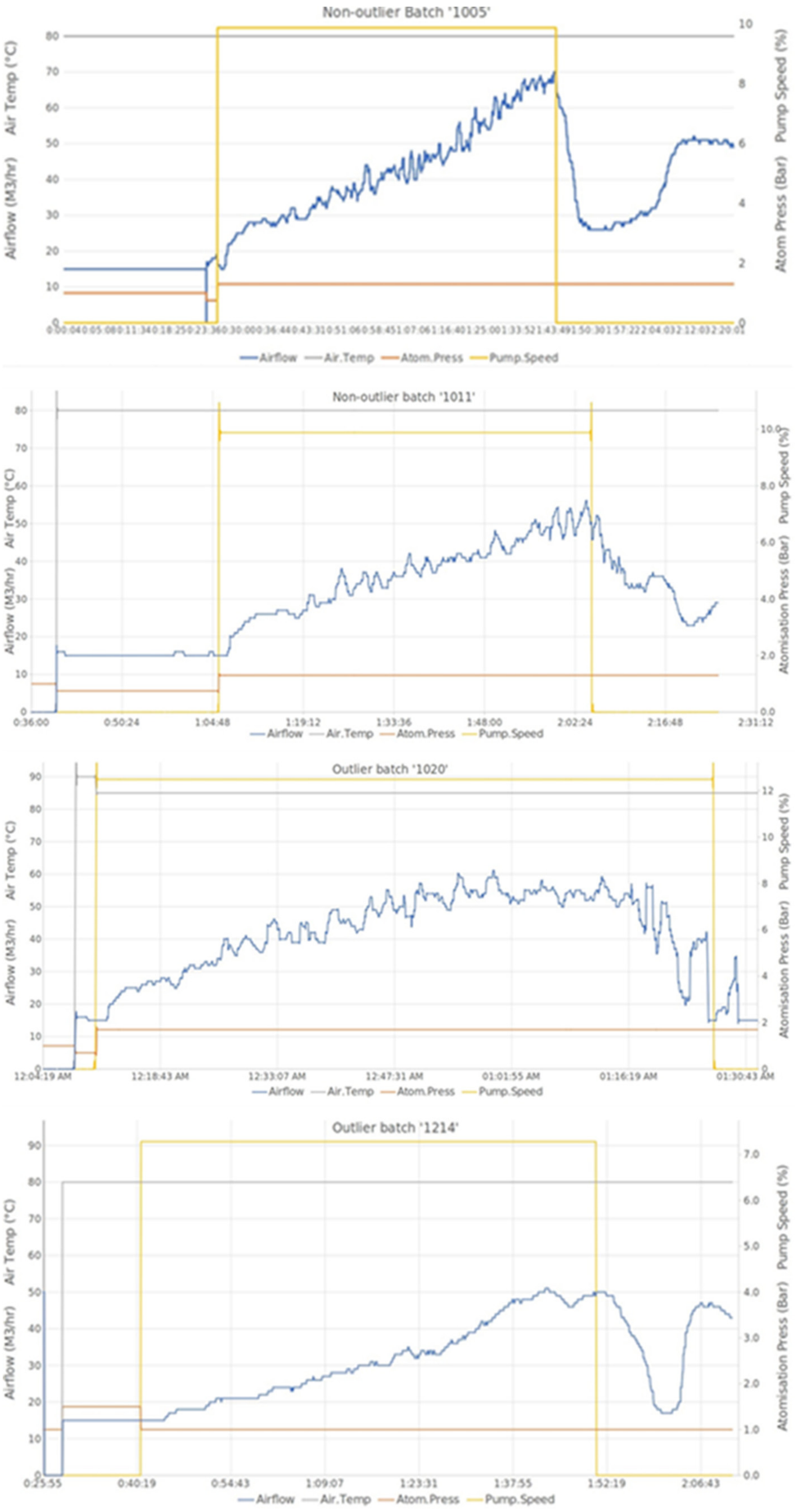

Figure S11. Airflow rate, Inlet Air Temperature, Atomization Pressure and Pump speed for two outlier and two non-outlier batches.

pharmaceutics

MDPI

Table S5. Granule size statistics (Dv10, Dv25, Dv75, Dv90) for 12 non-outlier batches, for Dv50, see Table 1.

Batch	Start-point	End-point	mean	st. dev.	min	max	range	Q3-Q1
Dv10	63.8	173.3	164.0	80.6	38.7	458.3	419.5	135.8
Dv25	87.5	229.0	222.8	107.9	45.0	610.9	565.9	148.6
Dv75	200.5	380.8	426.4	189.0	61.1	1008.9	947.8	281.1
Dv90	250.9	463.1	540.4	239.3	71.9	1119.7	1047.9	401.5

Table S6. Comparison in granule sizes external test RMSEP for MA 1 between NIR-PLS model and merged-PLS (NIR+ 17-PP PLS). Extended table of Table 2, and regards Figure 8.

	Dv10		Dv25		Dv50		Dv75		Dv90	
	**NIR**	**NIR+par**	**NIR**	**NIR+par**	**NIR**	**NIR+par**	**NIR**	**NIR+par**	**NIR**	**NIR+par**
20220802	25.3	18.8	24.1	18.9	19.7	14.8	18.9	21.1	25.3	28.1
20220920	84.5	10.3	106.5	9.58	123.3	10.8	147.0	18.7	203.8	30.8
20220930	42.0	34.5	33.1	23.9	45.0	23.2	64.2	30.9	77.2	29.0
20221005	117.4	61.3	152.7	69.6	179.8	68.6	205.6	59.7	259.2	121.0
20221011	46.6	39.3	38.0	48.4	47.3	23.9	102.0	83.5	149.6	121.8
20221020	61.9	101.9	119.4	140.9	197.6	208.9	288.9	337.0	441.5	538.1
20221128	43.5	16.4	53.7	27.0	42.7	98.7	44.7	185.6	55.1	272.7
20221129	19.2	14.2	29.1	17.4	30.5	27.4	58.0	22.6	74.3	18.5
20221203	80.5	27.1	92.9	50.2	134.9	84.1	189.0	63.2	264.3	45.8
20221212	79.5	58.2	95.4	49.6	91.4	26.6	84.5	95.6	108.2	174.2
20221213	31.7	33.1	29.2	32.7	29.1	34.8	32.3	26.7	35.3	15.3
20221214	139.7	85.0	183.8	94.6	251.7	136.4	315.8	234.7	346.6	282.3
20221229	56.3	26.0	64.0	20.5	43.6	39.0	35.4	126.0	54.1	209.7
20221230	17.6	25.1	28.4	19.0	32.3	13.6	25.7	21.1	27.3	25.9
**average**	60.4	39.4	75.0	44.4	90.6	57.9	115.1	94.7	151.6	136.7
**aver. -outliers**	53.7	30.4	62.3	32.2	68.3	38.8	83.9	62.9	111.1	91.1
T-test 1-t, p.	0.0118		0.0049		0.0171		0.1847		0.326	

Table S7. Comparison in granule sizes internal test RMSEP for MA 1 between NIR-PLS model and merged-PLS (NIR+ 17-PP PLS). See Figure 2 for difference between internal and external test sets.

	Dv10		Dv25		Dv50		Dv75		Dv90	
	NIR	NIR+par	NIR	NIR+par	NIR	NIR+par	NIR	NIR+par	NIR	NIR+par
20220802	9.41	8.24	11.1	9.02	13.7	11.7	29.6	25.0	43.5	39.1
20220920	17.2	10.4	19.9	11.4	21.5	13.1	30.8	22.9	53.3	44.1
20220930	11.7	11.7	15.0	20.3	25.6	27.1	38.5	40.6	48.1	50.5
20221005	20.5	13.9	27.9	17.5	40.9	23.1	52.1	35.3	73.8	52.6
20221011	13.8	8.95	16.0	11.7	25.6	17.3	42.4	31.2	56.1	44.4
20221020	33.1	25.0	36.9	25.8	54.5	34.3	80.5	51.7	105.6	84.2
20221128	16.7	13.5	24.0	18.4	37.0	27.4	54.5	41.1	65.9	48.9
20221129	13.1	10.5	19.1	14.5	28.9	23.9	42.0	34.0	47.6	40.3
20221203	10.1	7.12	15.0	8.77	27.1	11.2	34.5	15.5	39.6	25.8
20221212	10.8	8.53	14.0	9.76	21.4	16.7	33.2	24.9	32.6	30.3
20221213	7.63	7.27	11.9	9.21	13.4	10.0	11.9	10.5	14.2	12.7
20221214	14.0	11.9	20.1	18.3	27.3	23.7	29.7	25.1	31.9	26.0
20221229	10.2	9.14	13.3	11.4	20.4	16.7	28.6	24.6	33.4	31.4
20221230	10.1	7.78	11.4	8.87	17.2	14.3	21.6	19.7	30.2	28.8
average	14.2	11.0	18.3	13.9	26.8	19.3	37.9	28.7	48.3	39.9
aver. -outliers	12.6	9.8	16.6	12.6	24.4	17.7	35.0	27.1	44.9	37.4
T-test 1-t, p.	0.000193		0.000864		0.000411		0.000556		0.000659	

pharmaceutics MDPI

Table S8. Comparison in granule sizes of training set RMSECV for MA 1 between NIR-PLS model and merged-PLS (NIR+ 17-PP PLS). See Figure 2 related to first 90% of the data - training set (i.e. 81% of the data) for each separate batch (MA 1)

	Dv10		Dv25		Dv50		Dv75		Dv90	
	NIR	NIR+pars	NIR	NIR+pars	NIR	NIR+pars	NIR	NIR+pars	NIR	NIR+pars
20220802	9.49	7.89	11.8	8.98	13.5	12.0	28.8	26.0	45.4	43.1
20220920	17.2	10.6	20.8	11.5	23.0	13.6	32.3	23.8	53.5	45.2
20220930	11.8	11.5	15.2	15.1	25.5	26.6	37.6	41.0	45.7	55.7
20221005	20.6	15.3	28.0	19.4	41.2	26.3	55.3	39.2	81.9	55.0
20221011	15.3	9.57	17.5	12.2	27.9	18.5	45.8	32.0	58.9	45.2
20221020	33.0	26.0	36.9	26.4	53.4	34.4	78.9	52.3	105.2	80.8
20221128	16.6	13.2	24.1	17.8	34.5	26.2	52.9	39.5	67.7	49.8
20221129	13.1	12.6	19.1	17.7	28.5	27.7	41.5	38.3	47.1	43.6
20221203	10.7	7.51	15.3	9.11	28.3	11.9	36.0	16.4	40.4	28.3
20221212	10.7	7.90	13.3	9.52	20.7	15.5	32.4	23.8	34.5	28.6
20221213	7.88	7.73	11.2	9.21	12.0	9.97	11.7	10.4	14.1	13.3
20221214	14.1	10.2	20.8	15.3	27.5	19.5	30.4	22.5	30.5	23.0
20221229	11.3	9.59	14.8	12.6	22.7	18.5	31.3	26.7	33.9	30.7
20221230	10.8	9.05	12.3	10.5	17.8	15.3	23.0	21.1	32.7	29.2
average	14.5	11.3	18.7	14.0	26.9	19.7	38.4	29.5	49.4	40.8
aver. -outliers	13.0	10.2	17.0	12.8	24.6	18.5	35.7	28.2	46.3	39.0
T-test 1-t, p.	0.000105		5.36 x 10^-5		0.000403		0.000654		0.00301	

Table S9. Comparison in granule sizes of training set RMSEC for MA 1 between NIR-PLS model and merged-PLS (NIR+ 17-PP PLS). See Figure 2 related to first 90% of the data - training set (i.e. 81% of the data) for each separate batch (MA 1)

	Dv10		Dv25		Dv50		Dv75		Dv90	
	NIR	NIR+pars	NIR	NIR+pars	NIR	NIR+pars	NIR	NIR+pars	NIR	NIR+pars
20220802	9.14	7.72	11.4	8.75	12.8	11.6	27.4	25.3	43.4	42.0
20220920	16.6	10.4	19.8	11.3	21.9	13.4	31.0	23.5	51.7	44.7
20220930	11.5	11.2	14.7	14.7	24.7	26.0	36.3	40.0	44.1	54.8
20221005	19.9	15.1	27.1	19.1	39.7	25.8	53.7	38.6	79.5	54.7
20221011	15.0	9.46	16.9	12.0	27.2	18.3	44.7	31.8	57.1	44.8
20221020	32.6	25.6	36.5	26.2	52.9	34.1	78.3	52.0	104.5	80.3
20221128	15.2	12.6	22.4	16.9	32.1	25.1	49.1	37.6	62.8	47.7
20221129	12.3	12.1	18.0	17.0	26.8	26.8	38.9	37.3	45.1	42.8
20221203	10.1	7.42	14.3	8.90	26.8	11.5	33.8	16.1	38.0	27.8
20221212	10.3	7.79	12.8	9.34	19.3	15.2	30.2	23.3	30.9	27.9
20221213	7.60	7.61	10.6	9.08	11.1	9.56	10.9	9.90	13.1	12.9
20221214	14.0	10.0	20.5	15.0	27.2	19.1	30.0	22.1	30.0	22.6
20221229	11.3	9.59	14.8	12.6	22.4	18.5	30.5	26.5	32.3	30.3
20221230	10.4	8.83	11.9	10.2	17.2	14.8	21.8	20.5	31.1	28.4
average	14.0	11.1	18.0	13.6	25.9	19.3	36.9	28.9	47.4	40.1
aver. -outliers	12.4	10.0	16.2	12.5	23.5	18.0	34.0	27.5	44.1	38.2
T-test 1-t, p.	0.000191		7.74 x 10^-5		0.000679		0.00127		0.00685	

pharmaceutics

MDPI

Table S10. Comparison in granule sizes of training set RMSECV for MA 1 between NIR-PLS model and merged-PLS (NIR+ 17-PP PLS) using block-CV instead of sequential-CV (in Table S8).

	Dv10		Dv25		Dv50		Dv75		Dv90	
	NIR	NIR+pars	NIR	NIR+pars	NIR	NIR+pars	NIR	NIR+pars	NIR	NIR+pars
20220802	17.9	16.3	24.5	23.6	32.1	30.3	60.3	66.9	90.3	99.5
20220920	44.9	24.5	62.8	21.8	67.7	25.3	86.6	34.2	124.2	53.8
20220930	25.1	27.4	31.0	35.0	48.6	66.6	74.2	106.0	82.9	120.5
20221005	36.8	26.6	50.9	31.8	75.3	44.3	95.4	64.7	104.0	75.0
20221011	33.8	18.0	39.1	22.4	55.0	27.2	89.7	43.8	114.1	59.8
20221020	77.9	63.7	92.9	47.5	121.1	63.5	163.9	100.1	196.1	121.9
20221128	28.9	21.2	44.3	30.6	66.9	41.2	96.2	59.8	116.8	75.0
20221129	25.0	21.1	38.9	31.6	62.4	47.7	90.9	64.4	98.3	74.5
20221203	30.7	16.0	35.1	19.8	54.8	25.0	68.8	35.3	71.0	53.4
20221212	20.8	13.4	28.6	20.0	43.3	32.9	72.8	49.5	94.9	62.4
20221213	17.4	13.8	22.3	17.1	26.3	20.0	23.6	18.5	26.9	23.1
20221214	27.0	27.8	42.1	49.5	57.4	57.3	54.9	56.3	62.0	64.3
20221229	13.3	11.7	17.4	16.1	27.6	20.7	42.9	34.1	58.1	48.7
20221230	30.4	24.3	33.5	25.6	39.0	34.7	42.7	44.4	62.1	60.7
average	30.7	23.3	40.2	28.0	55.5	38.3	75.9	55.6	93.0	70.9
aver. -outliers	27.1	19.5	35.7	24.6	49.9	34.7	70.3	51.8	87.0	67.2
T-test 1-t, p.	0.00065		0.00508		0.00300		0.00613		0.01017	

T-test is calulated between averages of all 14 batches (i.e. not for aver. - outliers!).

Table S11. External test RMSEP statistic of MA 1 for NIR+pars: The same as Table S6, but NIR+ 18-PP PLS is here considered that includes spray rate as the 18th variables. 50%-l.s. represents MA 1-add with included spray rate process variables.

/percentage of	Dv10		Dv25		Dv50		Dv75		Dv90	
all batch data	NIR+pars	50%-l.s.	NIR+pars	50%-l.s.	NIR+pars	50%-l.s.	NIR+pars	50%-l.s.	NIR+pars	50%-l.s.
0802/14.7%	15.9	27.5	18.3	26.2	14.1	10.6	20.3	21.2	27.2	91.6
0920/20.8%	7.40	70.5	9.33	75.3	11.5	87.7	16.5	63.1	28.2	42.9
0930/26.8%	31.8	80.9	18.2	83.5	16.3	137.2	28.7	213.1	46.4	299.7
1005/13.0%	63.2	71.1	75.4	93.3	79.1	113.9	71.3	114.8	124.0	156.2
1011/8%	38.4	35.8	45.2	29.1	24.1	23.3	68.4	19.0	122.6	49.1
1020/6.9%	95.9	144.1	146.0	157.7	218.4	199.4	350.6	266.6	563.3	335.3
1128/7.9%	17.6	18.4	23.5	18.8	90.4	71.8	176.4	151.6	262.0	223.2
1129/10%	13.6	13.6	17.6	17.6	25.8	25.8	21.4	21.4	18.0	18.0
1203/4.3%	26.9	19.0	53.6	8.89	85.4	22.6	71.5	29.5	47.2	30.1
1212/10%	57.9	57.9	48.2	48.2	25.9	25.9	101.1	101.1	130.2	130.2
1213/24.1%	32.0	24.0	34.0	22.7	34.7	25.8	27.4	13.5	15.1	15.2
1214/6.5%	86.7	78.1	99.4	121.7	143.1	184.3	241.5	247.9	289.8	312.6
1229/10.3%	25.7	25.1	20.3	20.0	42.9	43.2	111.2	115.5	209.4	208.7
1230/16.3%	27.7	21.4	21.7	16.3	15.3	9.85	22.2	26.2	29.1	117.5
average	38.6	49.1	45.1	52.8	59.1	70.1	94.9	100.3	136.6	145.0
aver. -outliers	35.8	46.5	38.5	46.0	46.6	59.8	73.6	89.0	105.9	138.2

Table S12. Comparison in granule sizes prediction accuracy for MA 2c, i.e. for four-batch prediction data between NIR-PLS model and merged-PLS (NIR+ 17-PP PLS) (using eight other batches).

Test batches	NIR spectra only		NIR spec.+17 par.	
	RMSEP	R2te	RMSEP	R2te
1, 3, 7, 11	85.80	0.615	58.44	0.825
2, 5, 6, 9	133.4	0.430	79.50	0.663
4, 8, 10, 12	111.9	0.544	70.08	0.829
aver.	110.4	0.530	69.34	0.772

Table S13. Comparison in granule sizes external test RMSEP for MA 1, between NIR-PLS model and merged-PLS for NIR+ 16-PP PLS - without runtime.

	Dv10		Dv25		Dv50		Dv75		Dv90	
	NIR	NIR+pars	NIR	NIR+pars	NIR	NIR+pars	NIR	NIR+pars	NIR	NIR+pars
20220802	25.3	19.1	24.1	21.3	19.7	17.9	18.9	27.0	25.3	27.9
20220920	84.5	12.5	106.5	11.9	123.3	12.5	147.0	19.0	203.8	31.5
20220930	42.0	33.5	33.1	22.8	45.0	24.2	64.2	32.1	77.2	25.8
20221005	117.4	56.9	152.7	64.3	179.8	63.3	205.6	55.5	259.2	111.6
20221011	46.6	42.2	38.0	52.5	47.3	25.2	102.0	73.5	149.6	115.4
20221020	61.9	99.6	119.4	137.5	197.6	202.5	288.9	331.2	441.5	523.3
20221128	43.5	16.1	53.7	26.5	42.7	97.7	44.7	184.3	55.1	270.7
20221129	19.2	13.2	29.1	23.3	30.5	23.3	58.0	20.2	74.3	29.6
20221203	80.5	17.4	92.9	29.2	134.9	53.3	189.0	35.5	264.3	60.0
20221212	79.5	66.9	95.4	64.6	91.4	34.7	84.5	61.2	108.2	128.9
20221213	31.7	34.2	29.2	35.0	29.1	38.4	32.3	29.8	35.3	17.7
20221214	139.7	83.3	183.8	92.4	251.7	132.9	315.8	228.8	346.6	276.7
20221229	56.3	27.4	64.0	22.0	43.6	30.7	35.4	116.1	54.1	203.9
20221230	17.6	25.8	28.4	18.6	32.3	13.6	25.7	21.4	27.3	26.2
**average**	60.4	39.2	75.0	44.4	90.6	55.0	115.1	88.3	151.6	132.1
**aver. -outliers**	53.7	30.4	62.3	32.7	68.3	36.2	83.9	56.3	111.1	87.4
T-test 1-t, p.	0.0131		0.0062		0.0131		0.126		0.270	

Table S14. Comparison in granule sizes prediction accuracy for MA 2a, i.e. for three-batch prediction data between NIR-PLS model and merged-PLS (NIR+ 16-PP PLS) (using nine other batches) for the case of omitting runtime in PLS-modelling which led to somewhat weaker performance (as can be seen when compared to Table 3).

Test	NIR spectra only model				NIR spectra + 17 process par. model			
batches:	RMSECV	R2CV	RMSEP	R2te	RMSECV	R2CV	RMSEP	R2te
3, 7, 11	69.90	0.732	91.45	0.649	37.75	0.921	64.89	0.839
2, 5, 10	72.17	0.759	96.35	0.618	49.08	0.888	86.28	0.474
1, 6, 9	68.06	0.779	77.87	0.581	43.31	0.910	61.59	0.814
4, 8, 12	60.67	0.762	113.54	0.576	39.84	0.897	74.92	0.806
average	67.7	0.758	94.80	0.606	42.50	0.904	71.92	0.733

Table S15. Comparison in granule sizes prediction accuracy for MA 2b, i.e. for one-batch prediction data between NIR-PLS model and merged-PLS (NIR+ 16-PP PLS) (using eleven other batches) for the case of omitting runtime in PLS-modelling which led to somewhat weaker performance (as can be seen when compared to Table 4). Average Dv50 CV error for NIR spectra, CV = 71.95 um; for NIR+16 proc. parameters, CV = 43.62 um.

Test	NIR-only		NIR+16-pars	
batches	RMSEP	R2te	RMSEP	R2te
1	57.53	0.811	62.04	0.836
2	101.0	0.281	129.6	0.342
3	117.2	0.722	85.50	0.811
4	135.5	0.471	71.69	0.935
5	70.81	0.840	41.41	0.909
6	98.48	0.578	43.40	0.869
7	49.97	0.824	40.48	0.908
8	102.3	0.761	55.18	0.954
9	59.42	0.797	62.57	0.824
10	81.70	0.680	78.53	0.404
11	40.45	0.897	52.68	0.929
12	46.78	0.865	60.75	0.927
aver.	80.10	0.711	65.32	0.804

Table S16. Granule sizes external test RMSEP for MA 1 of Pars-only PLS model (17-pars) (result for comparison with Table S6)

	Dv10	Dv25	Dv50	Dv75	Dv90
20220802	18.7	15.2	18.0	32.5	76.3
20220920	8.04	13.0	14.3	23.9	41.0
20220930	13.9	26.5	85.6	141.4	173.3
20221005	71.0	93.8	106.9	109.4	160.0
20221011	31.2	25.5	51.3	93.8	171.4
20221020	73.3	195.0	270.2	357.4	557.4
20221128	28.1	60.5	137.8	244.4	313.5
20221129	6.77	10.5	37.7	77.5	121.7
20221203	45.9	69.8	138.8	223.8	271.6
20221212	38.3	41.8	60.1	128.8	208.3
20221213	21.6	33.3	37.6	23.9	11.2
20221214	97.8	129.8	185.8	308.1	381.7
20221229	40.1	47.0	132.7	247.0	352.6
20221230	24.0	32.6	35.8	34.9	44.5
**average**	37.1	56.7	93.8	146.2	206.0
**aver. -outliers**	29.0	39.1	71.4	115.1	162.1
T-test 1-t, p.*	0.281	0.0234	0.00012	0.00093	0.00079

* t-test between each of these columns and corresponding Table S6 column for NIR+pars PLS models.

Table S17. Granule sizes internal test RMSEP for MA 1 of Pars-only PLS model (17-PP) (result for comparison with Table S7)

	Dv10	Dv25	Dv50	Dv75	Dv90
20220802	9.72	12.3	17.2	33.9	53.9
20220920	11.4	12.5	14.5	26.2	49.6
20220930	15.0	21.5	42.9	67.0	74.0
20221005	16.7	22.0	28.8	41.9	52.5
20221011	11.2	14.1	19.8	32.0	46.2
20221020	45.8	35.1	37.5	56.2	92.4
20221128	15.8	20.7	29.4	44.1	54.7
20221129	13.3	17.5	30.8	44.5	64.1
20221203	9.45	11.2	11.3	24.1	40.9
20221212	8.81	9.54	15.6	28.0	35.4
20221213	10.1	10.1	12.4	13.3	19.2
20221214	14.8	22.7	29.5	32.8	38.3
20221229	11.1	13.2	19.9	31.8	39.5
20221230	11.1	12.8	20.1	26.2	40.2
**average**	14.6	16.8	23.6	35.9	50.1
**aver. -outliers**	12.0	14.8	21.9	34.4	47.5
T-test 1-t, p.*	0.0095	0.00022	0.00096	0.00044	7.9 x 10^-5

* t-test between each of these columns and corresponding Table S7 column for NIR+pars PLS models.

Table S18. Granule sizes RMSECV for MA 1 of Pars-only PLS model (17-PP) (result for comparison with Table S8)

	Dv10	Dv25	Dv50	Dv75	Dv90
20220802	9.80	12.1	18.0	35.0	58.9
20220920	12.0	12.7	14.8	26.8	50.5
20220930	16.9	24.1	47.2	73.6	82.8
20221005	18.3	24.4	31.4	44.0	53.8
20221011	11.6	14.5	20.8	31.8	46.3
20221020	46.0	35.6	37.9	56.9	89.0
20221128	15.7	20.7	29.3	44.9	56.5
20221129	15.1	20.7	34.1	49.8	64.3
20221203	9.46	11.7	12.8	24.0	40.7
20221212	9.17	10.8	16.7	29.7	36.1
20221213	9.86	9.91	12.1	13.0	19.6
20221214	13.2	20.2	26.3	30.1	36.5
20221229	11.0	13.5	20.7	32.3	37.9
20221230	12.3	15.1	23.8	31.9	47.3
**average**	15.0	17.6	24.7	37.4	51.4
**aver. -outliers**	12.6	15.9	23.5	36.4	49.6
T-test 1-t, p.*	0.0065	0.00012	0.0014	0.0011	0.00010

* t-test between each of these columns and corresponding Table S8 column for NIR+pars PLS models.

Table S19. Granule sizes RMSEC for MA 1 of Pars-only PLS model (17-PP) (result for comparison with Table S9)

	Dv10	Dv25	Dv50	Dv75	Dv90
20220802	9.64	12.1	17.9	35.0	58.8
20220920	11.9	12.2	14.6	26.6	50.1
20220930	16.7	23.7	46.7	72.9	81.9
20221005	18.2	24.2	31.1	43.8	53.6
20221011	11.5	14.5	20.7	31.7	46.2
20221020	45.9	35.5	37.9	56.8	88.9
20221128	15.6	20.6	29.1	44.6	56.1
20221129	14.9	20.4	33.9	49.6	58.6
20221203	9.35	11.5	12.7	23.9	40.5
20221212	9.12	10.7	16.6	29.5	36.0
20221213	9.86	9.81	12.0	12.9	19.6
20221214	13.2	20.1	26.2	30.0	36.5
20221229	11.0	13.5	20.7	32.3	37.7
20221230	12.3	14.9	23.6	31.1	46.4
**average**	14.9	17.4	24.6	37.2	50.8
**aver. -outliers**	12.5	15.7	23.3	36.2	48.8
T-test 1-t, p.*	0.0056	9.4 x 10^-5	0.00093	0.00077	5.6 x 10^-5

* t-test between each of these columns and corresponding Table S9 column for NIR+pars PLS models.

Table S20. MA 2a, RMSEP and R2te for MA 2a for Pars-only model. Results are comparable with Table 3, and are all repeated here for more clarity

Test	NIR spectra only		NIR spec.+17 par.		17 parms.	
batches	RMSEP	R2te	RMSEP	R2te	RMSEP	R2te
3, 7, 11	91.45	0.649	62.63	0.858	75.14	0.811
2, 5, 10	96.35	0.618	77.00	0.621	106.6	0.684
1, 6, 9	77.87	0.581	56.83	0.827	56.06	0.843
4, 8, 12	113.54	0.576	71.39	0.823	64.07	0.862
aver.	94.80	0.606	66.96	0.782	75.47	0.8

Comment: 12.7% rel diff. in RMSEP in favor of NIR+pars

Table S21. MA 2b, RMSEP and R2te for MA 2b for Pars-only model. Results are comparable with Table 4, and are all repeated here for more clarity

Test	NIR spectra only		NIR spec.+17 par.		17 parms.	
batches	RMSEP	R2te	RMSEP	R2te	RMSEP	R2te
1	57.53	0.811	64.19	0.861	57.18	0.774
2	101.0	0.281	86.99	0.446	84.77	0.402
3	117.2	0.722	76.50	0.876	94.75	0.788
4	135.5	0.471	67.64	0.934	49.41	0.953
5	70.81	0.840	33.88	0.947	33.77	0.946
6	98.48	0.578	41.31	0.884	44.81	0.904
7	49.97	0.824	40.82	0.906	40.75	0.908
8	102.3	0.761	49.97	0.961	40.63	0.964
9	59.42	0.797	54.10	0.847	56.72	0.830
10	81.70	0.680	87.88	0.457	36.53	0.752
11	40.45	0.897	62.94	0.921	88.55	0.841

12	46.78	0.865	43.95	0.947	56.68	0.898
aver.	80.10	0.711	59.18	0.832	57.05	0.83

Comment: 3.7 % rel diff in RMSEP in favor of pars.

Table S22. MA 2c, RMSEP and R2te for MA 2c for Pars-only model. Results are comparable with Table S12, and are all repeated here for more clarity

Test batches	NIR-only		NIR+pars		pars-only	
	RMSEP	R2te	RMSEP	R2te	RMSEP	R2te
1, 3, 7, 11	85.80	0.615	58.44	0.825	69.11	0.771
2, 5, 6, 9	133.4	0.430	79.50	0.663	103.2	0.600
4, 8, 10, 12	111.9	0.544	70.08	0.829	62.21	0.869
aver.	110.4	0.530	69.34	0.772	78.17	0.747

Comment: 12.7% rel diff. in RMSEP in favor of NIR+pars.

UVE-PLS code for MA 2a

```
library(baseline)
library(signal)
wavelength<-read.csv('wavelength.csv',sep='	',header=FALSE)

A<-read.csv("All_Process_param_0802.csv",sep=",",header=TRUE)
Parm1<-A[-1,]
Parm1<-Parm1[789:nrow(Parm1),]
Parm1<-data.matrix(Parm1)

A1<-read.csv("NIR_spectra_0802.csv",sep=",",header=FALSE)
Fe1<-A1[789:nrow(A1),]
Fe1<-data.matrix(Fe1)
B2_1<-as.vector(Parm1[,3])

A<-read.csv("All_Process_param_0920_II.csv",sep=",",header=TRUE)
Parm2<-A[-1,]
Parm2<-data.matrix(Parm2)
Parm2<-Parm2[2192:nrow(Parm2),]
A1<-read.csv("NIR_spectra_0920.csv",sep=",",header=FALSE)
Fe2<-A1[2192:nrow(A1),]
Fe2<-data.matrix(Fe2)
B2_2<-as.vector(Parm2[,3])


A<-read.csv("All_Process_param_0930_II.csv",sep=",",header=TRUE)
Parm3<-A[-1,]
Parm3<-data.matrix(Parm3)
A1<-read.csv("NIR_spectra_0930.csv",sep=",",header=FALSE)
Fe3<-data.matrix(A1)
Fe3<-data.matrix(Fe3)
B2_3<-as.vector(Parm3[,3])

A<-read.csv("All_Process_param_1005_II.csv",sep=",",header=TRUE)
Parm4<-A[-1,]
Parm4<-data.matrix(Parm4)
A1<-read.csv("NIR_spectra_1005.csv",sep=",",header=FALSE)
Fe4<-data.matrix(A1)
Fe4<-data.matrix(Fe4)
B2_4<-as.vector(Parm4[,3])

A<-read.csv("All_Process_param_1011_II.csv",sep=",",header=TRUE)
Parm5<-A[-1,]
Parm5<-Parm5[433:nrow(Parm5),]
Parm5<-data.matrix(Parm5)
A1<-read.csv("NIR_spectra_1011.csv",sep=",",header=FALSE)
Fe5<-A1[433:nrow(A1),]
Fe5<-data.matrix(Fe5)
B2_5<-as.vector(Parm5[,3])


A<-read.csv("All_Process_param_1128_II.csv",sep=",",header=TRUE)
Parm6<-A[-1,]
Parm6<-Parm6[195:nrow(Parm6),]
Parm6<-data.matrix(Parm6)
A1<-read.csv("NIR_spectra_1128.csv",sep=",",header=FALSE)
Fe6<-A1[195:nrow(A1),]
Fe6<-data.matrix(Fe6)
B2_6<-as.vector(Parm6[,3])

A<-read.csv("All_Process_param_1129_II.csv",sep=",",header=TRUE)
Parm7<-A[-1,]
Parm7<-Parm7[101:nrow(Parm7),]
Parm7<-data.matrix(Parm7)
A1<-read.csv("NIR_spectra_1129.csv",sep=",",header=FALSE)
Fe7<-A1[101:nrow(A1),]
Fe7<-data.matrix(Fe7)
B2_7<-as.vector(Parm7[,3])

A<-read.csv("All_Process_param_1203_II.csv",sep=",",header=TRUE)
Parm8<-A[-1,]
Parm8<-Parm8[152:nrow(Parm8),]
```

pharmaceutics MDPI

```
Parm8<-data.matrix(Parm8)
A1<-read.csv("NIR_spectra_1203.csv",sep=",",header=FALSE)
Fe8<-A1[152:nrow(A1),]
Fe8<-data.matrix(Fe8)
B2_8<-as.vector(Parm8[,3])

A<-read.csv("All_Process_param_1212_II.csv",sep=",",header=TRUE)
Parm9<-A[-1,]
Parm9<-data.matrix(Parm9)
A1<-read.csv("NIR_spectra_1212.csv",sep=",",header=FALSE)
Fe9<-data.matrix(A1)
Fe9<-data.matrix(Fe9)
B2_9<-as.vector(Parm9[,3])

A<-read.csv("All_Process_param_1213_II.csv",sep=",",header=TRUE)
Parm10<-A[-1,]
Parm10<-Parm10[621:nrow(Parm10),]
Parm10<-data.matrix(Parm10)
A1<-read.csv("NIR_spectra_1213.csv",sep=",",header=FALSE)
Fe10<-A1[621:nrow(A1),]
Fe10<-data.matrix(Fe10)
B2_10<-as.vector(Parm10[,3])

A<-read.csv("All_Process_param_1229_II.csv",sep=",",header=TRUE)
Parm11<-A[-1,]
Parm11<-data.matrix(Parm11)
A1<-read.csv("NIR_spectra_1229.csv",sep=",",header=FALSE)
Fe11<-data.matrix(A1)
Fe11<-data.matrix(Fe11)
B2_11<-as.vector(Parm11[,3])

A<-read.csv("All_Process_param_1230_II.csv",sep=",",header=TRUE)
Parm12<-A[-1,]
Parm12<-Parm12[679:nrow(Parm12),]
Parm12<-data.matrix(Parm12)
A1<-read.csv("NIR_spectra_1230.csv",sep=",",header=FALSE)
Fe12<-A1[679:nrow(A1),]
Fe12<-data.matrix(Fe12)
B2_12<-as.vector(Parm12[,3])

FFe1<-rbind(Fe1,Fe2,Fe3,Fe4,Fe5,Fe6,Fe7,Fe8,Fe9,Fe10,Fe11,Fe12)

Fe1<-FFe1

B2<-as.vector(t(cbind(t(B2_1),t(B2_2),t(B2_3),t(B2_4),t(B2_5),t(B2_6), t(B2_7), t(B2_8), t(B2_9), t(B2_10), t(B2_11), t(B2_12) ) ))

Vrum<-seq(1,length(B2),by=1)
# Calibration-Validation Experiment 1
Vtest1<-seq(3624,5426,by=1) # 3rd i.e. 0930
Vtest2<-seq(11967,13196,by=1) # 7th i.e. 1129
Vtest3<-seq(16288,17245,by=1) # 11th i.e. 1229

# Calibration-Validation Experiment 2
Vtest1<-seq(1201,3623,by=1) # 2nd i.e. 0920
Vtest2<-seq(8070,10425,by=1) # 5th i.e. 1011
Vtest3<-seq(15408,16287,by=1) # 10th i.e. 1213

# Calibration-Validation Experiment 3
Vtest1<-seq(1,1200,by=1) # 1st i.e. 0920
Vtest2<-seq(10426,11966,by=1) # 6th i.e. 1011
Vtest3<-seq(14464,15407,by=1) # 9th i.e. 1213

# Calibration-Validation Experiment 4
Vtest1<-seq(5427,8069,by=1) # 4th i.e. 1005
Vtest2<-seq(13197,14463,by=1) # 8th i.e. 1203
Vtest3<-seq(17246,18259,by=1) # 12th i.e. 1230

Vtest<-as.vector(cbind(t(Vtest1),t(Vtest2),t(Vtest3)))
Vtrain<-Vrum[-Vtest]

### Polyn. bas. corr
F<-Fe1
Q<-baseline.modpolyfit(F,degree = 4, tol = 0.001, rep = 100)
F<-Q$corrected
plot(Q$corrected[1,])
Fe1<-F
```

pharmaceutics

MDPI

```
### Polyn. bas. corr

SNV<-function(spectra){
  spectra<-as.matrix(spectra)
  spectrat<-t(spectra)
  spectrat_snv<-scale(spectrat,center=TRUE,scale=TRUE)
  spectra_snv<-t(spectrat_snv)
  return(spectra_snv)}
F<-SNV(Fe1)

Fe1<-F

Fe1_Parm1<-Parm1[,6:ncol(Parm1)]
Fe1_Parm2<-Parm2[,6:ncol(Parm2)]
Fe1_Parm3<-Parm3[,6:ncol(Parm3)]
Fe1_Parm4<-Parm4[,6:ncol(Parm4)]
Fe1_Parm5<-Parm5[,6:ncol(Parm5)]
Fe1_Parm6<-Parm6[,6:ncol(Parm6)]
Fe1_Parm7<-Parm7[,6:ncol(Parm7)]
Fe1_Parm8<-Parm8[,6:ncol(Parm8)]
Fe1_Parm9<-Parm9[,6:ncol(Parm9)]
Fe1_Parm10<-Parm10[,6:ncol(Parm10)]
Fe1_Parm11<-Parm11[,6:ncol(Parm11)]
Fe1_Parm12<-Parm12[,6:ncol(Parm12)]

Fe1_Parm<-
rbind(Fe1_Parm1,Fe1_Parm2,Fe1_Parm3,Fe1_Parm4,Fe1_Parm5,Fe1_Parm6,Fe1_Parm7,Fe1_Parm8,Fe1_Parm9,Fe1_Parm10,Fe1_Parm11,Fe1_Par
m12)


Fe_Parm<-Fe1_Parm[Vtrain,]
meanvect<-c()
sdvect<-c()
for (i in 1:ncol(Fe_Parm)) {
meanvect[i]<-mean(Fe_Parm[,i])
sdvect[i]<-sd(Fe_Parm[,i])}
for (i in 1:ncol(Fe1_Parm)) {
Fe1_Parm[,i]<- (Fe1_Parm[,i]- meanvect[i])/sdvect[i] }


Fe1<-cbind(F,Fe1_Parm)

F<-Fe1
B1<-B2[Vtrain]
Fe<-F[Vtrain,]

ncomp=8

kfold<-10
RMSECV<-array(ncomp)
PRESS<-array(ncomp)
Btest<-array(nrow(Fe))
Btrain<-array
xtrain<-matrix(rep(NA, (nrow(Fe)-1)*ncol(Fe)),nrow=(nrow(Fe)-1))
xtest<-matrix(rep(NA,1*ncol(Fe)),nrow=1)
B.pred<-matrix(rep(NA, ncomp*nrow(Fe)),nrow=ncomp)

for (m in 1:kfold) {
xtest<-Fe[seq(m,nrow(Fe),by=kfold),]
Btest[seq(m,nrow(Fe),by=kfold)]<-B1[seq(m,nrow(Fe),by=kfold)]
xtrain<-Fe[-seq(m,nrow(Fe),by=kfold),]
Btrain<-B1[-seq(m,nrow(Fe),by=kfold)]
Ftrain<-Btrain-mean(Btrain)
Etrain<-t(t(xtrain)-colMeans(xtrain))
Xb<- t(t(xtest)-colMeans(xtrain))
h<-Ftrain
T<-matrix(rep(NA, nrow(Etrain)*1),nrow=nrow(Etrain))
W<-matrix(rep(NA, ncol(Etrain)*1),nrow=ncol(Etrain))
P<-matrix(rep(NA, ncol(Etrain)*1),nrow=ncol(Etrain))
Q<-array(1)
for (i in 1:ncomp) {
S<-t(Etrain)%*%Ftrain
qp<-svd(S)
if (i==1) {
W[,i]<-qp$u
T[,i]<-Etrain%*%W[,i] }
```

```
else {
W<-cbind(W,qp$u)
T<-cbind(T,Etrain%*%W[,i]) }
ey<-t(T[,i])%*%T[,i]
ey1<-as.numeric(ey)
if (i==1) {
P[,i]<-t(Etrain)%*%T[,i]/ey1
Q[i]<-t(Ftrain)%*%T[,i]/ey1 }
else {
P<-cbind(P,t(Etrain)%*%T[,i]/ey1)
Q<-cbind(Q,t(Ftrain)%*%T[,i]/ey1) }
Etrain<-Etrain-T[,i]%*%t(P[,i])
Ftrain<-Ftrain-T[,i]%*%t(Q[i])
A<-solve(t(T)%*%T)%*%t(T)%*%h
R<-W%*%solve(t(P)%*%W)
B<-R%*%A
for (j in 1:length(seq(m,nrow(Fe),by=kfold))) {
B.pred[i,m+((j-1)*kfold)]<-Xb[j,]%*%B+t(t(mean(Btrain)))}
}
}
for (i in 1:ncomp) {
PRESS[i]<-0}

for (i in 1:ncomp) {
for (m in 1:nrow(Fe)) {
PRESS[i]<-PRESS[i]+(Btest[m]-B.pred[i,m])^2}
RMSECV[i]<-sqrt(PRESS[i]/(nrow(Fe)))}

ncomp<-which.min(RMSECV)


BB<- matrix(rep(NA,kfold*ncol(Fe)),nrow=kfold)

xtrain<-matrix(rep(NA, (nrow(Fe)-1)*ncol(Fe)),nrow=(nrow(Fe)-1))
for (i in 1:ncomp) {
PRESS[i]<-0}
for (m in 1:kfold) {
xtrain<-Fe[-seq(m,nrow(Fe),by=kfold),]
Btrain<-B1[-seq(m,nrow(Fe),by=kfold)]
Etrain<-t(t(xtrain)-colMeans(xtrain))
Ftrain<-Btrain-mean(Btrain)
lj<-Ftrain
T<-matrix(rep(NA, nrow(Etrain)*1),nrow=nrow(Etrain))
W<-matrix(rep(NA, ncol(Etrain)*1),nrow=ncol(Etrain))
P<-matrix(rep(NA, ncol(Etrain)*1),nrow=ncol(Etrain))
Q<-array(1)
for (i in 1:ncomp) {
S<-t(Etrain)%*%Ftrain
qp<-svd(S)
if (i==1) {
W[,i]<-qp$u
T[,i]<-Etrain%*%W[,i] }
else {
W<-cbind(W,qp$u)
T<-cbind(T,Etrain%*%W[,i]) }
ey<-t(T[,i])%*%T[,i]
ey1<-as.numeric(ey)
if (i==1) {
P[,i]<-t(Etrain)%*%T[,i]/ey1
Q[i]<-t(Ftrain)%*%T[,i]/ey1 }
else {
P<-cbind(P,t(Etrain)%*%T[,i]/ey1)
Q<-cbind(Q,t(Ftrain)%*%T[,i]/ey1) }
Etrain<-Etrain-T[,i]%*%t(P[,i])
Ftrain<-Ftrain-T[,i]%*%t(Q[i])
A<-solve(t(T)%*%T)%*%t(T)%*%lj
R<-W%*%solve(t(P)%*%W)
}
B<-R%*%A
BB[m,]<-t(B)
for (i in 1:ncol(BB)) {
BB[m,i]<-abs(BB[m,i])}
 skala<-BB[m,which.max(BB[m,])]
 BB[m,]<-BB[m,]/skala
}
```

```
division<-20
Feg<-F
dim(F)
Stdev<-array(ncol(BB))
Me<-array(ncol(BB))
Tvalue<-array(ncol(BB))
for (i in 1:ncol(BB)) {
Me[i]<-mean(BB[,i])
Stdev[i]<-0
for (j in 1:nrow(BB)) {
Stdev[i]<-Stdev[i]+((BB[j,i]-Me[i])^2)}
Stdev[i]<-sqrt(Stdev[i]/(nrow(BB)-1))
Tvalue[i]<-Me[i]/Stdev[i]
if (NaN %in% Tvalue[i]) {
Tvalue[i]<-0.1}}

zon<-sort(Tvalue,decreasing=TRUE)
pon<-trunc(zon[ncomp+2])
step<-pon/division
ploz<-array(division)
ploz1<-array(division)
for (h in 1:division) {
brojac<-0
Zerd<-c()
Bvar<-c()
for (i in 1:ncol(Feg)) {
if (Tvalue[i]>(h*step)) {
brojac<-brojac+1
if (brojac==1) {
Zerd<-Feg[,i]
Bvar<-i}
else {
Zerd<-cbind(Zerd,Feg[,i])
Bvar<-cbind(Bvar,i)}}
}
Bvar<-as.vector(Bvar)

F<-Zerd
B1<-B2[Vtrain]
Fe<-F[Vtrain,]

B.predd<-matrix(rep(NA, ncomp*nrow(Fe)),nrow=ncomp)
kfold<-10
RMSECV<-array(ncomp)
PRESS<-array(ncomp)
Btest<-array(nrow(Fe))
Btrain<-array
xtrain<-matrix(rep(NA, (nrow(Fe)-1)*ncol(Fe)),nrow=(nrow(Fe)-1))
xtest<-matrix(rep(NA,1*ncol(Fe)),nrow=1)
B.pred<-matrix(rep(NA, ncomp*nrow(Fe)),nrow=ncomp)

for (m in 1:kfold) {
xtest<-Fe[seq(m,nrow(Fe),by=kfold),]
Btest[seq(m,nrow(Fe),by=kfold)]<-B1[seq(m,nrow(Fe),by=kfold)]
xtrain<-Fe[-seq(m,nrow(Fe),by=kfold),]
Btrain<-B1[-seq(m,nrow(Fe),by=kfold)]
Ftrain<-Btrain-mean(Btrain)
Etrain<-t(t(xtrain)-colMeans(xtrain))
Xb<- t(t(xtest)-colMeans(xtrain))
lj<-Ftrain
T<-matrix(rep(NA, nrow(Etrain)*1),nrow=nrow(Etrain))
W<-matrix(rep(NA, ncol(Etrain)*1),nrow=ncol(Etrain))
P<-matrix(rep(NA, ncol(Etrain)*1),nrow=ncol(Etrain))
Q<-array(1)
for (i in 1:ncomp) {
S<-t(Etrain)%*%Ftrain
qp<-svd(S)
if (i==1) {
W[,i]<-qp$u
T[,i]<-Etrain%*%W[,i] }
else {
W<-cbind(W,qp$u)
T<-cbind(T,Etrain%*%W[,i]) }
ey<-t(T[,i])%*%T[,i]
ey1<-as.numeric(ey)
if (i==1) {
```

```
P[,i]<-t(Etrain)%*%T[,i]/ey1
Q[i]<-t(Ftrain)%*%T[,i]/ey1 }
else {
P<-cbind(P,t(Etrain)%*%T[,i]/ey1)
Q<-cbind(Q,t(Ftrain)%*%T[,i]/ey1) }
Etrain<-Etrain-T[,i]%*%t(P[,i])
Ftrain<-Ftrain-T[,i]%*%t(Q[i])
A<-solve(t(T)%*%T)%*%t(T)%*%lj
R<-W%*%solve(t(P)%*%W)
B<-R%*%A
for (j in 1:length(seq(m,nrow(Fe),by=kfold))) {
B.pred[i,m+((j-1)*kfold)]<-Xb[j,]%*%B+t(t(mean(Btrain)))}
}
}
for (i in 1:ncomp) {
PRESS[i]<-0}

for (i in 1:ncomp) {
for (m in 1:nrow(Fe)) {
PRESS[i]<-PRESS[i]+(Btest[m]-B.pred[i,m])^2}
RMSECV[i]<-sqrt(PRESS[i]/(nrow(Fe)))}

ploz[h]<-RMSECV[which.min(RMSECV)]
}

h<-which.min(ploz)

brojac<-0
Zerd<-c()
Bvar<-c()
for (i in 1:ncol(Feg)) {
if (Tvalue[i]>(h*step)) {
brojac<-brojac+1
if (brojac==1) {
Zerd<-Feg[,i]
Bvar<-i}
else {
Zerd<-cbind(Zerd,Feg[,i])
Bvar<-cbind(Bvar,i)}}
}
Bvar<-as.vector(Bvar)

F<-Zerd

dim(F)

B1<-B2[Vtrain]
Fe<-F[Vtrain,]




B.predd<-matrix(rep(NA, ncomp*nrow(Fe)),nrow=ncomp)
kfold<-10
RMSECV<-array(ncomp)
PRESS<-array(ncomp)
Btest<-array(nrow(Fe))
Btrain<-array
xtrain<-matrix(rep(NA, (nrow(Fe)-1)*ncol(Fe)),nrow=(nrow(Fe)-1))
xtest<-matrix(rep(NA,1*ncol(Fe)),nrow=1)
B.pred<-matrix(rep(NA, ncomp*nrow(Fe)),nrow=ncomp)

for (m in 1:kfold) {
xtest<-Fe[seq(m,nrow(Fe),by=kfold),]
Btest[seq(m,nrow(Fe),by=kfold)]<-B1[seq(m,nrow(Fe),by=kfold)]
xtrain<-Fe[-seq(m,nrow(Fe),by=kfold),]
Btrain<-B1[-seq(m,nrow(Fe),by=kfold)]
Ftrain<-Btrain-mean(Btrain)
Etrain<-t(t(xtrain)-colMeans(xtrain))
Xb<- t(t(xtest)-colMeans(xtrain))
lj<-Ftrain
T<-matrix(rep(NA, nrow(Etrain)*1),nrow=nrow(Etrain))
W<-matrix(rep(NA, ncol(Etrain)*1),nrow=ncol(Etrain))
P<-matrix(rep(NA, ncol(Etrain)*1),nrow=ncol(Etrain))
Q<-array(1)
```

```
for (i in 1:ncomp) {
S<-t(Etrain)%*%Ftrain
qp<-svd(S)
if (i==1) {
W[,i]<-qp$u
T[,i]<-Etrain%*%W[,i] }
else {
W<-cbind(W,qp$u)
T<-cbind(T,Etrain%*%W[,i]) }
ey<-t(T[,i])%*%T[,i]
ey1<-as.numeric(ey)
if (i==1) {
P[,i]<-t(Etrain)%*%T[,i]/ey1
Q[i]<-t(Ftrain)%*%T[,i]/ey1 }
else {
P<-cbind(P,t(Etrain)%*%T[,i]/ey1)
Q<-cbind(Q,t(Ftrain)%*%T[,i]/ey1) }
Etrain<-Etrain-T[,i]%*%t(P[,i])
Ftrain<-Ftrain-T[,i]%*%t(Q[i])
A<-solve(t(T)%*%T)%*%t(T)%*%lj
R<-W%*%solve(t(P)%*%W)
B<-R%*%A
for (j in 1:length(seq(m,nrow(Fe),by=kfold))) {
B.pred[i,m+((j-1)*kfold)]<-Xb[j,]%*%B+t(t(mean(Btrain)))}
}
}
for (i in 1:ncomp) {
PRESS[i]<-0}

for (i in 1:ncomp) {
for (m in 1:nrow(Fe)) {
PRESS[i]<-PRESS[i]+(Btest[m]-B.pred[i,m])^2}
RMSECV[i]<-sqrt(PRESS[i]/(nrow(Fe)))}

ncomp<-which.min(RMSECV)



xtrain<-F[Vtrain,]
xtest<-F[Vtest,]
Btrain<-B2[Vtrain]
Btest<-B2[Vtest]


X1<-mean(Btrain)
Ftrain<-array(length(Btrain))
for (i in 1:length(Btrain)) {
Ftrain[i]<-Btrain[i]-X1}
X<-colMeans(xtrain)
for (i in 1:nrow(xtrain)) {
if (i==1) {
X2<-X}
else {
X2<-rbind(X2,X) }}
Etrain<-xtrain-X2
Etr<-Etrain
for (hj in 1:length(Btest)) {
if (hj==1) {
X2<-X }
else {
X2<-rbind(X2,X)}}
Xb<-xtest-X2
lj<-Ftrain
T<-matrix(rep(NA, nrow(Etrain)*1),nrow=nrow(Etrain))
W<-matrix(rep(NA, ncol(Etrain)*1),nrow=ncol(Etrain))
P<-matrix(rep(NA, ncol(Etrain)*1),nrow=ncol(Etrain))
Q<-array(1)
for (i in 1:ncomp) {
S<-t(Etrain)%*%Ftrain
qp<-svd(S)
if (i==1) {
W[,i]<-qp$u
T[,i]<-Etrain%*%W[,i] }
else {
W<-cbind(W,qp$u)
T<-cbind(T,Etrain%*%W[,i]) }
```

```
ey<-t(T[,i])%*%T[,i]
ey1<-as.numeric(ey)
if (i==1) {
P[,i]<-t(Etrain)%*%T[,i]/ey1
Q[i]<-t(Ftrain)%*%T[,i]/ey1 }
else {
P<-cbind(P,t(Etrain)%*%T[,i]/ey1)
Q<-cbind(Q,t(Ftrain)%*%T[,i]/ey1) }
Etrain<-Etrain-T[,i]%*%t(P[,i])
Ftrain<-Ftrain-T[,i]%*%t(Q[i])
}
A<-solve(t(T)%*%T)%*%t(T)%*%lj
R<-W%*%solve(t(P)%*%W)
B<-R%*%A
B.pred.train<-array(length(Btrain))
B.pred.test<-array(length(Btest))
B.predd.train<-array(length(Btrain))
B.predd.test<-array(length(Btest))
PCCtrain<-0
PCCtest<-0


for (i in 1:length(Btrain)) {
B.pred.train[i]<-Etr[i,]%*%B+t(t(X1))
if (B.pred.train[i]>0.5) {
B.predd.train[i]<-1}
else {
B.predd.train[i]<-0}
if (B.predd.train[i]==Btrain[i]) {
PCCtrain<-PCCtrain+1/length(Btrain)}
}


for (i in 1:length(Btest)) {
B.pred.test[i]<-Xb[i,]%*%B+t(t(X1))
if (B.pred.test[i]>0.5) {
B.predd.test[i]<-1}
else {
B.predd.test[i]<-0}
if (B.predd.test[i]==Btest[i]) {
PCCtest<-PCCtest+1/length(Btest)}
}



ncomp
RMSEC<-sqrt(sum((B.pred.train-Btrain)^2)/(nrow(Fe)-1-ncomp))
RMSEP<-sqrt(sum((B.pred.test-Btest)^2)/(length(Btest)))



k1<-B.pred.train
k<-Btrain

a<-array(length(k1))
for (i in 1:length(k1)) {
a[i]<-k1[i]}
k1<-a
L<-length(k)
e<-matrix(rep(NA,2,nrow=2))
x1<- matrix(rep(NA, L*2),nrow= L)
y1<- matrix(rep(NA, L),nrow= L)
for (i in 1:length(k)) {
for(j in 1:2) {
x1[i,j]<-k1[i]^(j-1)

}
}

z<-solve(t(x1)%*%x1)%*%t(x1)
for (i in 1:length(k1)) {
y1[i,1]<-k[i]
}
e<-z%*%y1
w<-array(length(k))
for (i in 1:length(k)) {
```

pharmaceutics

```
w[i]<-(x1[i,2]*e[2,1])+e[1,1] }
w
f<-0
for (i in 1:length(k)) {
f<-f+y1[i,1]}
f<-f/length(k)

x11<-matrix(rep(NA, length(k)*2),nrow= length(k))
for (i11 in 1:length(k)) {
for(j11 in 1:2) {
x11[i11,j11]<-w[i11]^(j11-1)}
}
z11<-solve(t(x11)%*%x11)%*%t(x11)
e11<-z11%*%y1
Ttest.pred2<-array(length(k))
for (i in 1:length(k)) {
Ttest.pred2[i]<-(x11[i,2]*e11[2,1])+e11[1,1] }

g<-0
g1<-0
for (i in 1:length(k)) {
g<-g+(y1[i,1]-f)*(y1[i,1]-f)
g1<-g1+(y1[i,1]-Ttest.pred2[i])*(y1[i,1]-Ttest.pred2[i]) }
r2tr<-1-(g1/g)
r2tr


k1<-B.pred[ncomp,]
k<-Btrain

a<-array(length(k1))
for (i in 1:length(k1)) {
a[i]<-k1[i]}
k1<-a
L<-length(k)
e<-matrix(rep(NA,2,nrow=2))
x1<- matrix(rep(NA, L*2),nrow= L)
y1<- matrix(rep(NA, L),nrow= L)
for (i in 1:length(k)) {
for(j in 1:2) {
x1[i,j]<-k1[i]^(j-1)

}
}

z<-solve(t(x1)%*%x1)%*%t(x1)
for (i in 1:length(k1)) {
y1[i,1]<-k[i]
}
e<-z%*%y1
w<-array(length(k))
for (i in 1:length(k)) {
w[i]<-(x1[i,2]*e[2,1])+e[1,1] }
w
f<-0
for (i in 1:length(k)) {
f<-f+y1[i,1]}
f<-f/length(k)

x11<-matrix(rep(NA, length(k)*2),nrow= length(k))
for (i11 in 1:length(k)) {
for(j11 in 1:2) {
x11[i11,j11]<-w[i11]^(j11-1)}
}
z11<-solve(t(x11)%*%x11)%*%t(x11)
e11<-z11%*%y1
Ttest.pred2<-array(length(k))
for (i in 1:length(k)) {
Ttest.pred2[i]<-(x11[i,2]*e11[2,1])+e11[1,1] }

g<-0
g1<-0
for (i in 1:length(k)) {
g<-g+(y1[i,1]-f)*(y1[i,1]-f)
g1<-g1+(y1[i,1]-Ttest.pred2[i])*(y1[i,1]-Ttest.pred2[i]) }
r2cv<-1-(g1/g)
```

```
r2cv


k1<-B.pred.test
k<-Btest
a<-array(length(k1))
for (i in 1:length(k1)) {
a[i]<-k1[i]}
k1<-a
L<-length(k)
e<-matrix(rep(NA,2,nrow=2))
x1<- matrix(rep(NA, L*2),nrow= L)
y1<- matrix(rep(NA, L),nrow= L)
for (i in 1:length(k)) {
for(j in 1:2) {
x1[i,j]<-k1[i]^(j-1)

}
}

z<-solve(t(x1)%*%x1)%*%t(x1)
for (i in 1:length(k1)) {
y1[i,1]<-k[i]
}
e<-z%*%y1
w<-array(length(k))
for (i in 1:length(k)) {
w[i]<-(x1[i,2]*e[2,1])+e[1,1] }
w
f<-0
for (i in 1:length(k)) {
f<-f+y1[i,1]}
f<-f/length(k)

x11<-matrix(rep(NA, length(k)*2),nrow= length(k))
for (i11 in 1:length(k)) {
for(j11 in 1:2) {
x11[i11,j11]<-w[i11]^(j11-1)}
}
z11<-solve(t(x11)%*%x11)%*%t(x11)
e11<-z11%*%y1
Ttest.pred2<-array(length(k))
for (i in 1:length(k)) {
Ttest.pred2[i]<-(x11[i,2]*e11[2,1])+e11[1,1] }

g<-0
g1<-0
for (i in 1:length(k)) {
g<-g+(y1[i,1]-f)*(y1[i,1]-f)
g1<-g1+(y1[i,1]-Ttest.pred2[i])*(y1[i,1]-Ttest.pred2[i]) }
r2te<-1-(g1/g)
r2te


ncomp
RMSEC
RMSEP
r2tr
r2te
RMSECV[which.min(RMSECV)]
r2cv
dim(F)
```
